# Evolutionary Analysis of the *VP1* and RNA-Dependent RNA Polymerase Regions of Human Norovirus GII.P17-GII.17 in 2013–2017

**DOI:** 10.3389/fmicb.2019.02189

**Published:** 2019-09-27

**Authors:** Yuki Matsushima, Fuminori Mizukoshi, Naomi Sakon, Yen Hai Doan, Yo Ueki, Yasutaka Ogawa, Takumi Motoya, Hiroyuki Tsukagoshi, Noriko Nakamura, Naoki Shigemoto, Hideaki Yoshitomi, Reiko Okamoto-Nakagawa, Rieko Suzuki, Rika Tsutsui, Fumio Terasoma, Tomoko Takahashi, Kenji Sadamasu, Hideaki Shimizu, Nobuhiko Okabe, Koo Nagasawa, Jumpei Aso, Haruyuki Ishii, Makoto Kuroda, Akihide Ryo, Kazuhiko Katayama, Hirokazu Kimura

**Affiliations:** ^1^Division of Virology, Kawasaki City Institute for Public Health, Kawasaki, Japan; ^2^Department of Microbiology, Tochigi Prefectural Institute of Public Health and Environmental Science, Utsunomiya, Japan; ^3^Department of Microbiology, Osaka Institute of Public Health, Osaka, Japan; ^4^Department of Virology II, National Institute of Infectious Diseases, Musashimurayama, Japan; ^5^Department of Microbiology, Miyagi Prefectural Institute of Public Health and Environment, Sendai, Japan; ^6^Division of Virology, Saitama Institute of Public Health, Saitama, Japan; ^7^Ibaraki Prefectural Institute of Public Health, Mito, Japan; ^8^Gunma Prefectural Institute of Public Health and Environmental Sciences, Maebashi, Japan; ^9^Aichi Prefectural Institute of Public Health, Kita, Japan; ^10^Hiroshima Prefectural Technology Research Institute Public Health and Environment Center, Hiroshima, Japan; ^11^Fukuoka Institute of Health and Environmental Sciences, Dazaifu, Japan; ^12^Yamaguchi Prefectural Institute of Public Health and Environment, Yamaguchi, Japan; ^13^Kanagawa Prefectural Institute of Public Health, Chigasaki, Japan; ^14^Aomori Prefecture Public Health and Environment Center, Aomori, Japan; ^15^Wakayama Prefectural Research Center of Environment and Public Health, Wakayama, Japan; ^16^Iwate Prefectural Research Institute for Environmental Sciences and Public Health, Morioka, Japan; ^17^Department of Microbiology, Tokyo Metropolitan Institute of Public Health, Shinjuku, Japan; ^18^Eastern Chiba Medical Center, Togane, Japan; ^19^Graduate School of Health Sciences, Gunma Paz University, Takasaki, Japan; ^20^Department of Respiratory Medicine, Kyorin University School of Medicine, Mitaka, Japan; ^21^Pathogen Genomics Center, National Institute of Infectious Diseases, Musashimurayama, Japan; ^22^Department of Microbiology, Yokohama City University, Graduate School of Medicine, Yokohama, Japan; ^23^Laboratory of Viral Infection I, Kitasato Institute for Life Sciences, Graduate School of Infection Control Sciences, Kitasato University, Minato, Japan

**Keywords:** GII.P17-GII.17, human norovirus, molecular evolution, RdRp, VP1

## Abstract

Human norovirus (HuNoV) GII.P17-GII.17 (Kawasaki2014 variant) reportedly emerged in 2014 and caused gastroenteritis outbreaks worldwide. To clarify the evolution of both *VP1* and RNA-dependent RNA polymerase (*RdRp*) regions of GII.P17-GII.17, we analyzed both global and novel Japanese strains detected during 2013–2017. Time-scaled phylogenetic trees revealed that the ancestral GII.17 *VP1* region diverged around 1949, while the ancestral GII.P17 *RdRp* region diverged around 2010. The evolutionary rates of the *VP1* and *RdRp* regions were estimated at ~2.7 × 10^−3^ and ~2.3 × 10^−3^ substitutions/site/year, respectively. The phylogenetic distances of the *VP1* region exhibited no overlaps between intra-cluster and inter-cluster peaks in the GII.17 strains, whereas those of the *RdRp* region exhibited a unimodal distribution in the GII.P17 strains. Conformational epitope positions in the VP1 protein of the GII.P17-GII.17 strains were similar, although some substitutions, insertions and deletions had occurred. Strains belonging to the same cluster also harbored substitutions around the binding sites for the histo-blood group antigens of the VP1 protein. Moreover, some amino acid substitutions were estimated to be near the interface between monomers and the active site of the RdRp protein. These results suggest that the GII.P17-GII.17 virus has produced variants with the potential to alter viral antigenicity, host-binding capability, and replication property over the past 10 years.

## Introduction

Human norovirus (HuNoV) is a major causative pathogen of acute viral gastroenteritis (de Graaf et al., 2016). Although HuNoV is most prevalent during autumn and winter in the northern hemisphere, the virus can be detected throughout the year (Ahmed et al., 2013). A previous report suggested that approximately 680 million people annually suffer from gastroenteritis due to HuNoV infection worldwide (Kirk et al., 2015). Molecular epidemiological studies have indicated that distinct GII.4 variants have recurrently emerged every 2–3 years in the past decade to cause pandemics of gastroenteritis in all aged individuals (Bull et al., 2010). Furthermore, GII.P17-GII.17 (Kawasaki2014 variant) has suddenly been prevalent since 2014 in various countries including Japan, China, South Korea, Italy, Romania, Argentina, Brazil, and the USA (Chan et al., 2015; Fu et al., 2015; Matsushima et al., 2015; Medici et al., 2015; Dang Thanh et al., 2016; Dinu et al., 2016; Cannon et al., 2017; Degiuseppe et al., 2017; Silva et al., 2017). However, the epidemiology of this virus over a long period of time is unclear at present.

HuNoV belongs to the genus *Norovirus* of the family *Caliciviridae*, and it is further genetically classified into three genogroups, GI, GII, and GIV. Genetic analyses have shown that HuNoV GI and GII can be further classified into 9 and 22 genotypes, respectively (Kroneman et al., 2013). Of these, many genotypes have been associated with gastroenteritis outbreaks throughout the world (Hoa Tran et al., 2013). The genomes of noroviruses contain three open reading frames and encodes six non-structural proteins, including the RNA-dependent RNA polymerase and the two capsid proteins VP1 and VP2 (de Graaf et al., 2016).

Molecular evolutionary analysis based on advanced bioinformatics technologies is a powerful tool to better understand not only the phylogeny of pathogens, but also their antigenicity (Bok et al., 2009; Siebenga et al., 2010; Boon et al., 2011; Lu et al., 2016; Parra et al., 2017; Tohma et al., 2017; Nagasawa et al., 2018). Furthermore, next-generation sequencing technologies are also useful for the comprehensive analysis of various virus genomes (Quiñones-Mateu et al., 2014). In this study, we used both these methodologies to conduct a detailed molecular evolutionary analysis of the *VP1* and *RdRp* regions of GII.P17-GII.17 strains detected in various countries.

## Materials and Methods

### Sample Preparation and Ethics Statement

A total of 76 strains of GII.P17-GII.17 detected in Miyagi (16 strains), Kanagawa (11 samples), Saitama (10 samples), Ibaraki (9 strains), Gunma (7 strains), Aichi (7 strains), Hiroshima (5 strains), Tochigi (4 strains), Fukuoka (3 strains), Yamaguchi (3 strains), and Aomori (1 strain) prefectures from 2013 to 2017 were sequenced in this study. Fecal samples were collected from patients with acute gastroenteritis associated with HuNoV infection under compliance with the Food Sanitation Law and the Law Concerning the Prevention of Infections and Medical Care for Patients of Infections of Japan. Informed consent was obtained from all participants, which was acquired from the subjects or their legally acceptable representatives for sample donation. The personal data of the patients was anonymized. To perform extraneous study (this study) and due to the lack of written informed consent, this study obtained ethical approval from the Research and Ethical Committees for the Use of Human Subjects of the National Institute of Infectious Diseases, Tokyo, Japan (No. 576). All methods were conducted in accordance with the approved guidelines. Information on the samples is given in Table S1. RNA was extracted from 10% suspensions of fecal samples in phosphate buffered saline using a QIAamp Viral RNA Mini kit (Qiagen, Hilden, Germany). The extracted RNA was subjected to sequencing as described below.

### Sequencing

Sequencing was performed with Sanger and next-generation sequencers. For Sanger sequencing, a reverse transcription–polymerase chain reaction (RT-PCR) was first performed for 30 min at 45°C and then 2 min at 94°C, followed by a total of 45 cycles of 30 s at 98°C, 30 s at 55°C and 90 s at 68°C, and then a final extension of 7 min at 68°C using specific primers for the *VP1* and *RdRp* regions and a PrimeScript II High Fidelity One Step RT-PCR kit (TaKaRa, Shiga, Japan; Table S2). Cycle sequencing was performed for 1 min at 96°C, followed by a total of 30 cycles of 10 s at 96°C, 10 s at 50°C and 2 min at 60°C using a BigDye Terminator v3.1 Cycle Sequencing kit (Applied Biosystems, Carlsbad, California, USA). The DNA sequences were analyzed using a 3500 Genetic Analyser (Applied Biosystems). Full-length nucleotide sequences of the *VP1* and *RdRp* regions were acquired using the primer walking method. Next-generation sequencing was conducted as described previously (Dennis et al., 2014; Ide et al., 2015). Data analysis was performed using CLC Genomics Workbench v8.0.1 (Qiagen). Contigs were assembled from the obtained sequence reads by *de novo* assembly. HuNoV genotypes were determined using the Norovirus Genotyping Tool (version 2.0) and the Human Calicivirus Typing Tool^1^ (Kroneman et al., 2011).

### Construction of Datasets for Bioinformatics

All full-length nucleotide sequences of the *VP1* and *RdRp* regions of GII.17, including information on sample collection years and no mixed nucleotides, were obtained from GenBank^2^ (accessed on 29 August, 2017). For the GII.P17-GII.17 genotype, only sequences with information on sample collection years and months were used in this study. Moreover, nine sequences associated with some recent outbreaks of GII.P17-GII.17 (Sakon et al., 2018) were combined with those of the new Japanese strains above. To construct time-scaled phylogenetic tree, we added representative *VP1* sequences of all GII genotypes, including porcine NoV GII (GII.11, GII.18, and GII.19) and other HuNoV GII genotypes (18 strains), as well as an outgroup strain of HuNoV GI genotype (GI.1) to the dataset of the *VP1* region (resulting in a total of 365 strains). The representative *RdRp* sequences of the GII genotypes, including porcine NoV (GII.P11 and GII.P18) and other HuNoV GII genotypes (20 strains), as well as an outgroup strain of HuNoV GI genotype (GI.P1), were appended to produce an *RdRp* dataset consisting of 156 strains (Table S1). The constructed datasets were aligned with MAFFT software (Katoh and Standley, 2013).

### Time-Scaled Phylogenetic Trees

Phylogenetic trees with molecular clocks were generated by the Bayesian Markov Chain Monte Carlo (MCMC) method using the BEAST2 package v2.4.8 (Bouckaert et al., 2014). Best substitution models (GTR+Γ+I for both *VP1* and *RdRp*) were determined for the constructed datasets (365 strains for *VP1* and 156 strains for the *RdRp*) by comparison of the Bayesian Information Criterion (BIC) values using jModelTest2 software (Guindon and Gascuel, 2003; Darriba et al., 2012). Appropriate clock and tree prior models (relaxed clock exponential and coalescent exponential population tree prior for both *VP1* and *RdRp*) were selected by path-sampling/stepping stone-sampling marginal-likelihood estimation using the BEAST2 package (Baele et al., 2012). The MCMC runs were conducted with chain lengths of 930,000,000 steps with sampling every 16,000 steps for *VP1*, and with chain lengths of 465,000,000 steps with sampling every 8,000 steps for *RdRp*. The analyzed data was evaluated by the effective sample sizes (ESS) values using Tracer^3^ software, and values of 200 or more were accepted. Maximum clade credibility trees were generated by discarding the first 10% of trees (burn-in) using TreeAnnotator v2.4.8 in the BEAST2 package. The time-scaled phylogenetic trees were visualized by FigTree^4^ v1.4.0 software. Branch reliability was supported by highest posterior densities (HPDs) of 95%. Moreover, the evolutionary rates of GII.P17-GII.17 strains were estimated as described above. The analyzed parameters with substitution, clock, and tree prior models are shown in Table 1. In this study, the evolutionary rates of overall clusters in the GII.P17-GII.17 strains were calculated since the rate of a cluster could not be estimated due to the limited number of strains available for analysis.

**Table 1:** Evolutionary rates of GII.P17-GII.17 strains in the *VP1* and *RdRp* regions.

**Regions**	**Numbers of strains**	**Substitution models**	**Clock models**	**Tree prior models**	**Evolutionary rates (95% HPD intervals)**
*VP1*	337	SYM+Γ	Strict	Constant size	2.7 × 10^−3^ (2.2–3.2 × 10^−3^)
*RdRp*	133	K80+Γ	Relaxed exponential	Exponential	2.3 × 10^−3^ (1.5–3.2 × 10^−3^)

### Bayesian Skyline Plot

Transition of effective population sizes was estimated by a Bayesian skyline plot using the BEAST2 package v2.4.8. Appropriate substitution models were selected based on comparison of the BIC values in the dataset, including 337 strains of the Kawasaki2014 variant in the *VP1* region. The clock models were appropriately selected on the basis of path-sampling/stepping stone-sampling marginal-likelihood estimation. The MCMC was run on chain lengths of 480,000,000 steps with sampling every 2,000 steps. After evaluation based on the ESS values, the Bayesian skyline plot was generated by Tracer^3^ software.

### Phylogenetic Distances

Phylogenetic trees were created using the maximum likelihood method in MEGA7 software (Kumar et al., 2016). Best substitution models (GTR+Γ+I for *VP1* and K80+Γ for *RdRp*) were selected using the jModelTest2. Branch reliability was supported by 1,000 replications of bootstrap values. Phylogenetic distances between GII.17 strains were calculated by Patristic software (Fourment and Gibbs, 2006).

### Construction of Three-Dimensional Structures, Conformational Epitope Analyses, and Selective Pressure Analyses

Structural models of the VP1 and RdRp proteins of HuNoV [Hu/GII/JP/1976/GII.17/Tokyo/27-3: AB684681 (VP1), Hu/GII/NE/1995/GII.P3/Amsterdam/1: KJ194500 (RdRp), Hu/GII/JP/2014/GII.P17-GII.17/Kawasaki323: AB983218 (VP1 and RdRp), Hu/GII/JP/2015/GII.P17-GII.17/Kawasaki308: LC037415 (VP1 and RdRp)] were generated based on homology modeling using the MODELLER software v9.15 (Webb and Sali, 2014). Three crystal structures for VP1 (PDBID: 1IHM, 5F4M and 5LKC) and one for RdRp (PDBID: 1SH0) were used as the templates based on Basic Local Alignment Search Tool^5^ (BLAST) analyses of the proteins. Amino acid sequences of the templates and the target strains were aligned using MAFFTash software (Standley et al., 2007; Katoh et al., 2009). The constructed structures were minimized using the GROMOS96, implemented by Swiss PDB Viewer v4.1 and evaluated by Ramachandran plots via the RAMPAGE server, which showed the favored regions of 96.1% ± 0.26 (VP1) and 98.0% ± 0.46 (RdRp), the allowed regions of 3.43% ± 0.32 (VP1) and 1.47% ± 0.32 (RdRp), and the outlier regions of 0.47% ± 0.058 (VP1) and 0.57% ± 0.15 (RdRp) (mean ± SD) of all residues in each structure (Guex and Peitsch, 1997; Lovell et al., 2003). The final models were modified and colored using Chimera v1.12 (Pettersen et al., 2004). Conformational epitopes on the VP1 dimer structures of the GII.17 strains were predicted using DiscoTope 2.0, EPCES, and EPSVR programmes (Liang et al., 2007, 2010; Kringelum et al., 2012). Cut-off values were set at −3.1 for the DiscoTope 2.0 and 81 for the EPCES and EPSVR in order to encompass epitopes on the VP1 of GII.17 identified by a previous *in vitro* study (Lindesmith et al., 2017). Consensus sites identified by more than one of the three tools and regions with two or more closely apposed residues of the sites on the VP1 dimer structures were determined to be conformational epitopes. Positive selection sites in the *VP1* region of the GII.17 strains were predicted using the Fast, Unconstrained Bayesian AppRoximation (FUBAR) and Mixed Effects Model of Evolution (MEME) algorithms on the Datamonkey server (Pond and Frost, 2005; Delport et al., 2010; Murrell et al., 2012, 2013; Weaver et al., 2018). Generally, FUBAR hypothesizes constant selection pressure at each site for the entire phylogeny and utilizes a Bayesian approach to estimate non-synonymous (*dN*) and synonymous (*dS*) substitution rates at each site. MEME is used to detect positive selection sites under a proportion of branches. Significance levels were set at posterior probabilities of >0.9 for FUBAR and *p* < 0.05 for MEME.

### Median Joining Network Analyses

Transmission links between GII.P17-GII.17 strains were analyzed using a median joining network with PopART software (Bandelt et al., 1999; Leigh and Bryant, 2015). We constructed a dataset that covered nucleotide sequence lengths from the start position of *RdRp* to the terminal position of *VP1* (resulting in a total of 114 strains). The dataset was then processed to detect recombination between the *RdRp* and *VP1* sequences based on seven primary exploratory recombination signal detection methods (RDP, GENECONV, BootScan, MaxChi, Chimera, SiScan, and 3Seq) using RDP4 software (Martin et al., 2015) and the threshold of the *p*-value for significance was set at 0.001. Recombination regions were assigned when they were identified by more than four of the seven methods; however, this criterion resulted in the identification of no recombinant sequences in the dataset. The median joining networks were analyzed using an epsilon value of zero.

## Results

### Time-Scaled Phylogeny of the *VP1* and *RdRp* Regions in the GII.P17-GII.17 Strains

**Figure 1:**
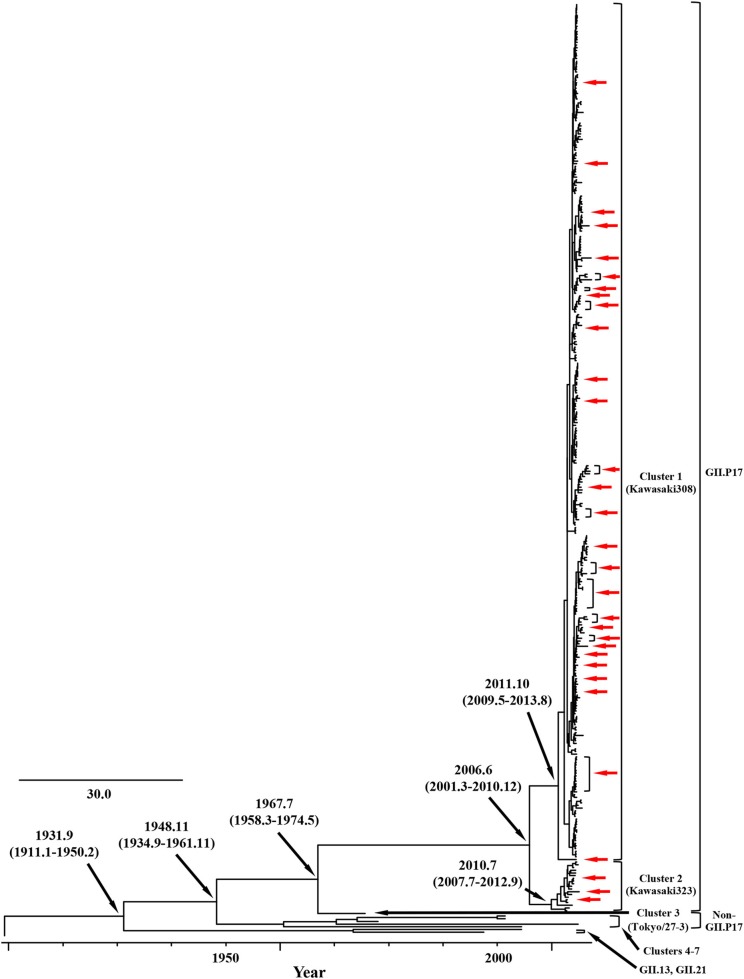
Time-scaled phylogenetic trees of the full-length *VP1* region of HuNoV constructed using the Bayesian MCMC inference. The visualization of the tree is enlarged to focus on GII.17, GII.13, and GII.21. The values within parentheses indicate the 95% HPDs for each divergent year, and red arrows indicate the Japanese samples collected in this study.

**Figure 2:**
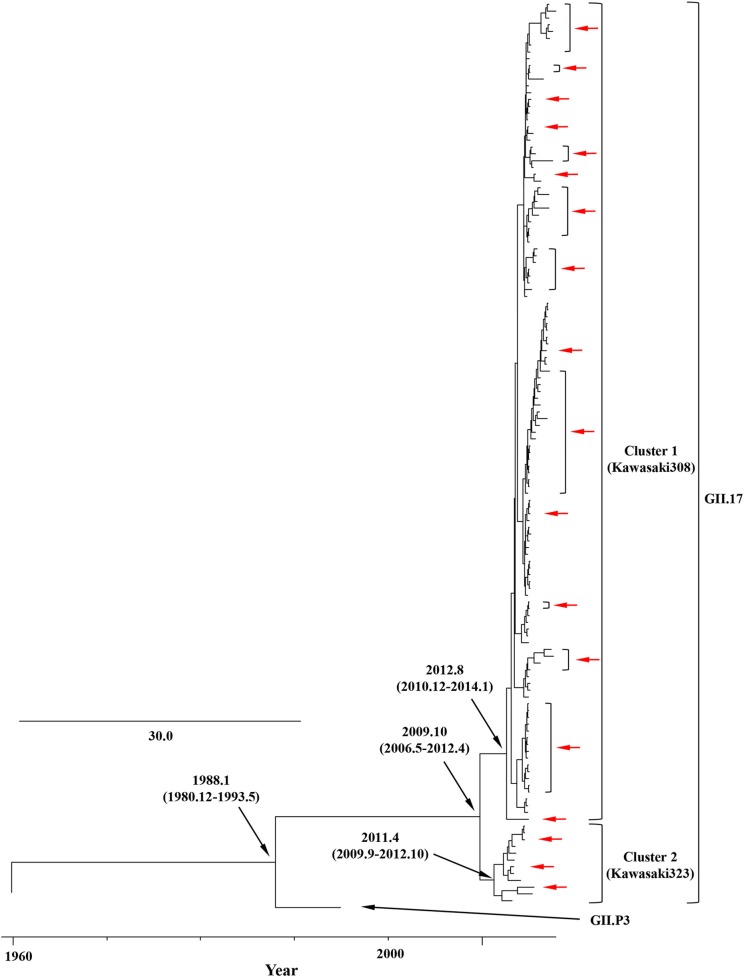
Time-scaled phylogenetic trees of the full-length *RdRp* region of HuNoV constructed using the Bayesian MCMC inference. The tree visualization is enlarged to focus on GII.P17 and GII.P3. The values within parentheses indicate the 95% HPDs for each divergent year, and red arrows indicate the Japanese samples collected in this study.

**Figure 3:**
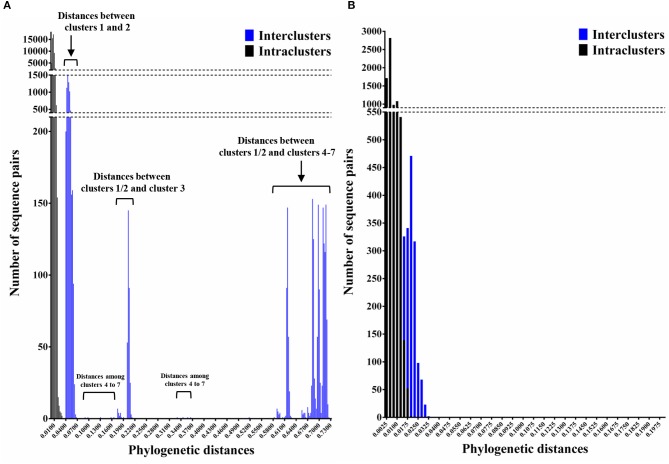
Phylogenetic distances between the nucleotide sequences of GII.17 strains in the full-length *VP1* and *RdRp* regions. **(A)** Distribution of intra-genotype phylogenetic distances in the *VP1* region of the GII.17 strains; **(B)** Distribution of intra-genotype phylogenetic distances in the *RdRp* region of the GII.P17 strains. The y-axis shows the number of sequence pairs corresponding to each distance while the x-axis represents phylogenetic distances.

We constructed time-scaled phylogenetic trees using the MCMC method based on the full-length of the *VP1* (343 strains) and *RdRp* (133 strains) regions in the GII.17 strains detected in the various countries (Figures 1, 2). The MCMC tree for the *VP1* region estimated that the common ancestor of the GII.17, GII.13, and GII.21 diverged in September, 1931 (95% HPD January, 1911–February, 1950). A common ancestor of the GII.17 strains diverged in November, 1948 (95% HPD September, 1934–November, 1961) and further diverged into seven clusters by around October, 2011. Of these, the GII.P17-GII.17 strains belonged to clusters 1 and 2 (Figure 1). The GII.17 strains in clusters 3, 4, 5, and 6 (no information on the *RdRp* sequence in cluster 7) were composed of the distinct *RdRp* genotypes, including GII.P3, GII.P16, GII.P4, and GII.Pe, respectively (Figure 1 and Table S1). Most strains of the GII.P17-GII.17 belonged to the cluster 1 (the Kawasaki308 type). The common ancestor of the cluster 1 diverged in October, 2011 (95% HPD May, 2009–August, 2013), while that of cluster 2 (the Kawasaki323 type) emerged in July, 2010 (95% HPD July, 2007–September, 2012) (Figure 1). Moreover, analyses of phylogenetic distances exhibited no overlap between intra- and inter-cluster peaks at a value of 0.035 (Figure 3A). With respect to the *RdRp* region (Figure 2), the common ancestor of the GII.P17 and GII.P3 diverged in January, 1988 (95% HPD December, 1980–May, 1993). Subsequently, the common ancestor of the GII.P17 diverged in October, 2009 (95% HPD May, 2006–April, 2012) and then further into two clusters by around August, 2012. The GII.P17-GII.17 strains were contained in clusters 1 and 2 in the *RdRp* region, which was compatible with the classification in the *VP1* region. Most strains of the GII.P17-GII.17 also belonged to cluster 1 (the Kawasaki308 type). The common ancestor of cluster 1 diverged in August, 2012 (95% HPD December, 2010–January, 2014), while that of cluster 2 (the Kawasaki323 type) did so in April, 2011 (95% HPD September, 2009–October, 2012). Furthermore, phylogenetic distances overlapped between the intra- and inter-cluster peaks (Figure 3B). These results suggest that the GII.P17-GII.17 strains emerged and formed two variants in approximately the past 10 years.

### Evolutionary Rates of the *VP1* and *RdRp* Regions in the GII.P17-GII.17 Strains

The evolutionary rates of the *VP1* and *RdRp* regions of the GII.P17-GII.17 strains are presented in Table 1. The rate for the *VP1* region was 2.7 × 10^−3^ substitutions/site/year (95% HPD 2.2–3.2 × 10^−3^), while that for the *RdRp* region was 2.3 × 10^−3^ (95% HPD 1.5–3.2 × 10^−3^). These results indicate that the *VP1* and *RdRp* regions of the GII.P17-GII.17 strains evolved with similar rates.

### Phylodynamics of GII.P17-GII.17 Strains in the *VP1* Region

**Figure 4:**
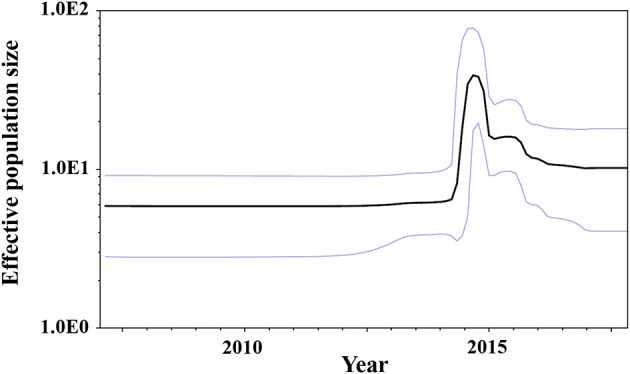
Bayesian skyline plots for the *VP1* sequences of GII.P17-GII.17 strains. The y-axis represents the effective population sizes on a logarithmic scale, whereas the x-axis denotes time in years. The solid black line indicates the mean posterior value and the intervals with 95% HPDs are shown by blue lines.

We estimated the transition of population sizes of GII.P17-GII.17 in the *VP1* region using a Bayesian skyline plot. The population of this region increased sharply in around 2014 and remained constant from 2016 after an immediate reduction (Figure 4). These results suggest that this was compatible between the fluctuation of the plot and actually epidemiological GII.17 prevalence.

### Prediction of Conformational Epitopes and Selective Pressures in the VP1 in GII.P17-GII.17 Strains

**Figure 5:**
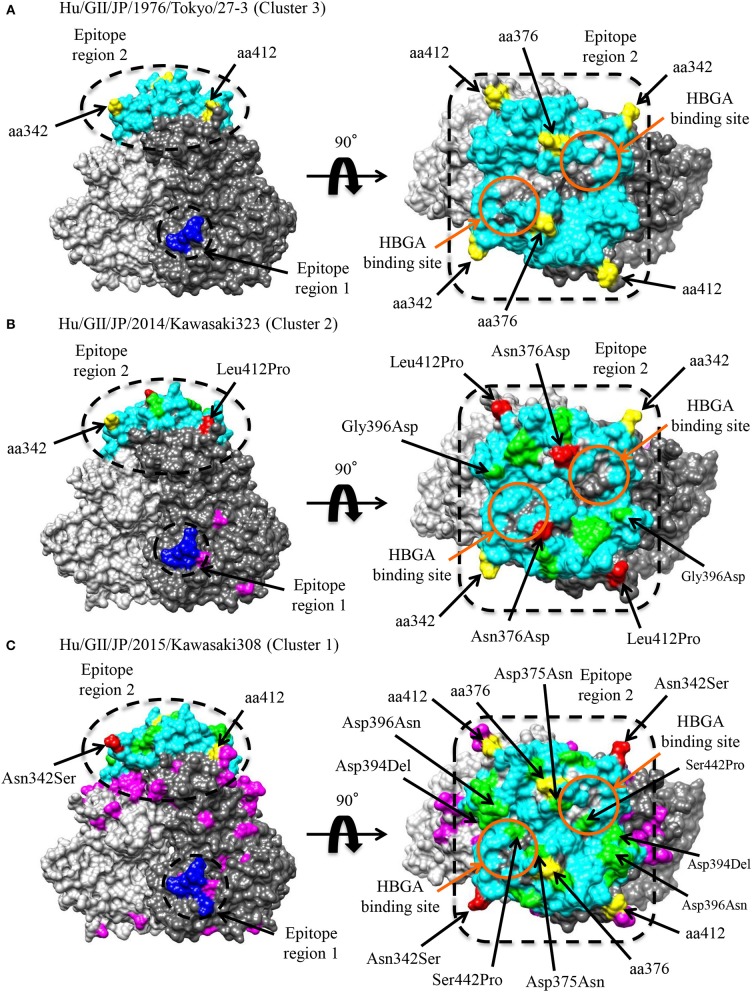
Structural models of the VP1 protein for representative GII.17 strains in the clusters of the Kawasaki2014 variant and its ancestor. Three-dimensional VP1 dimer structures for Tokyo/27-3 [ancestor; cluster 3; **(A)**], Kawasaki323 [cluster 2; **(B)**], and Kawasaki308 [cluster 1; **(C)**] viruses are shown. Each monomeric chain that comprise the dimer structures is colored gray (chain A) and dim gray (chain B). The predicted epitope regions of the strains are circled in black and the amino acids of the epitopes with no substitutions are colored blue for the shell domain and cyan for the P2 domain. Positive selection sites are colored yellow with the positions under the order of alignments. Orange circles represent the HBGA binding sites on the structures. Amino acid substitutions on the epitopes, non-epitopes, and positive selection sites within the clusters are colored green, magenta, and red, respectively.

Conformational epitopes and selective pressures in the VP1 protein were analyzed in order to assess the possibility of antigenicity changes at pre and post emergence of GII.P17-GII.17 based on the time-scaled phylogeny for the *VP1* region in Figure 1. As a result, common epitopes among strains of clusters 1, 2 (GII.P17-GII.17 clusters) and 3 [a closely related cluster (GII.P3-GII.17 strain) to the GII.P17-GII.17 clusters] were found in the shell domain and the protruding 2 (P2) domain. Although the amino acid epitope positions were similar among the strains, many amino acid substitutions were identified (Figure 5 and Table 2). Substitutions in epitopes were also identified in strains belonging to the same cluster. Of these substitution residues, amino acids (aa) 375, aa376, aa394, aa396, and aa442 are located close to the histo-blood group antigen (HBGA) binding sites of VP1, which are utilized for attachment of HuNoV to host cells. Additionally, three positive selection sites were identified in the P2 domain (Asn342Ser, Glu376Asn/Asp, and Arg412Leu/Glu/Pro). Of these sites, aa342 and aa376 were at predicted epitopes (Figure 5). These results indicate that GII.P17-GII.17 could evolve with changes in antigenicity and binding affinity to HBGAs.

**Table 2:** Conformational epitopes of GII.17 variants in the VP1 protein.

**Epitope region**		**Region 1**						**Region 2**																												
Amino acid positions^†^		163	168	170	171	172	173	289	291	292	293	294	295	296	297	298	299	300	301	328	329	330	339	340	341	342	343	344	345	346	347	351	352	354	358	359
Previously identified epitopes^#^																																				
Positive selection sites																																				
Predicted epitopes^$^	Hu/GII/JP/1976/Tokyo/27-3 (Cluster 3)	N	Y	N	Q	P	A	R	T	A	E	T	D	N	P	D	K	W	H	R	G	L	N	V	G	N	D	A	P	G	S	H	E	V	T	S
	Hu/GII/JP/2014/Kawasaki323 (Cluster 2)	.	.	.	.	.	N	.	.	.	.	.	.	H	R	.	.	.	.	K	.	V	.	.	.	.	.	.	.	.	.	.	.	.	Y	.
	Hu/GII/JP/2015/Kawasaki308 (Cluster 1)	.	.	.	.	.	N	.	.	.	Q	I	N	Q	R	.	R	.	.	K	.	V	.	.	.	.	.	.	.	.	.	Q	Q	W	Y	.
**Epitope region**		**Region 2**																																		
Amino acid positions^†^		360	361	372	374	375	376	377	378	379	380	381	382	383	392	393	394	395	396	397	398	399	400	401	402	403	405	442	443	444	445	446	447	448	449	450
Previously identified epitopes^#^																																				
Positive selection sites^§^																																				
Predicted epitopes^$^	Hu/GII/JP/1976/Tokyo/27-3 (Cluster 3)	P	Q	R	T	S	E	-	D	F	R	T	G	Q	I	N	D	E	D	N	-	H	P	F	N	Q	E	S	G	G	Y	N	Q	G	I	I
	Hu/GII/JP/2014/Kawasaki323 (Cluster 2)	.	.	.	N	D	N	-	.	.	Q	L	-	.	.	.	.	D	G	D	-	.	.	.	R	.	.	.	.	.	.	.	.	.	.	V
	Hu/GII/JP/2015/Kawasaki308 (Cluster 1)	.	.	.	S	D	N	D	.	.	Q	F	-	.	V	.	.	D	.	D	G	.	.	.	R	.	.	.	.	.	.	.	.	.	.	.

† *Amino acid positions are presented under the alignments*.

# *Blue indicates epitopes identified by Lindesmith et al. (2017)*.

§ *Green indicates positive selection sites predicted in this study*.

$ *Red indicates conformational epitopes predicted in this study*.

### Mapping of Amino Acid Substitutions of GII.P17-GII.17 to the RdRp Protein

**Figure 6:**
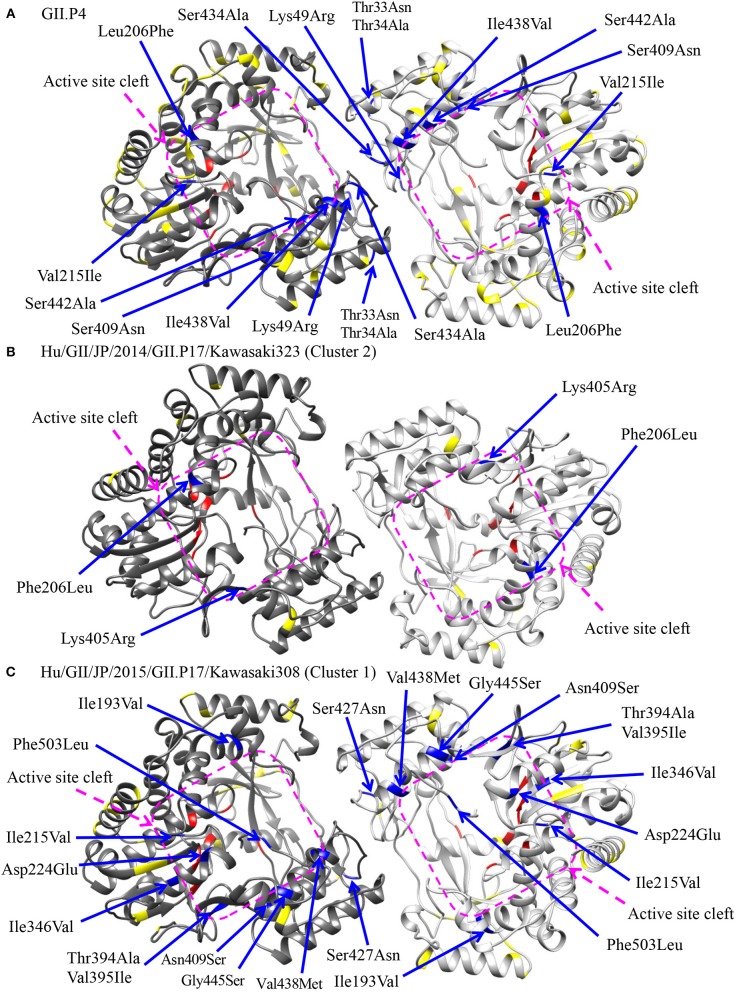
Structural models of the RdRp protein for representative GII.P17 strains. Homodimer structures of the RdRp of GII.P4 [PDBID 1SH0; **(A)**], Kawasaki323 [cluster 2; **(B)**], and Kawasaki308 [cluster 1; **(C)**] are shown. Each monomeric chain that compose the dimer structure are colored gray (chain A) and dim gray (chain B). Amino acid substitutions between GII.P17 and GII.P4 **(A)** or within the GII.P17 clusters **(B,C)** that are close to the interface between monomers and to the active site cleft are colored blue with the positions under the order of alignments, whereas the substitutions distant from these regions are shown in yellow. The residues of active sites for RNA replication are colored red. The purple quadrilaterals highlight the region of the active site cleft.

To assess the possibility of changes of RNA replication properties at pre and post emergence of GII.P17-GII.17 based on the time-scaled phylogeny for the *RdRp* region in Figure 2, we mapped the amino acid substitutions between GII.P17 strains and other *RdRp* genotypes onto three-dimensional structures of the RdRp protein (Figure 6). A total of 48 amino acid substitutions were identified among the GII.P17 and GII.P4 strains (Accession number; FJ537137). Of these, the substitutions at aa33, aa34, aa49, and aa434 were located close to the interface between monomers, while substitutions at aa206, aa215, aa409, aa438, and aa442 were located close to the active site (Figure 6A). Moreover, a total of 16 amino acid substitutions were estimated among the GII.P17 and GII.P3 (a closely related genotype to GII.P17) strains (Accession number; KJ194500). The substitution at aa49 was located adjacent to the interface between monomers, whereas the substitutions at aa206, aa215, aa407, and aa409 were located proximal to the active site (Figure S1). For intra-GII.P17 genotype, Phe206Leu and Lys405Arg substitutions were found around the active site in the strains of cluster 2 (12 strains) (Figure 6B). Thirty one substitutions were also found in the strains belonging to the cluster 1 (121 strains), and a Ser427Asn substitution was located proximally at the interface between monomers. Ile193Val, Ile215Val, Asp224Glu, Ile346Val, Thr394Ala, Val395Ile, Asn409Ser, Val438Met, Gly445Ser, and Phe503Leu substitutions were located close to the active sites (Figure 6C).

### Transmission Network Between the GII.P17-GII.17 Strains

**Figure 7:**
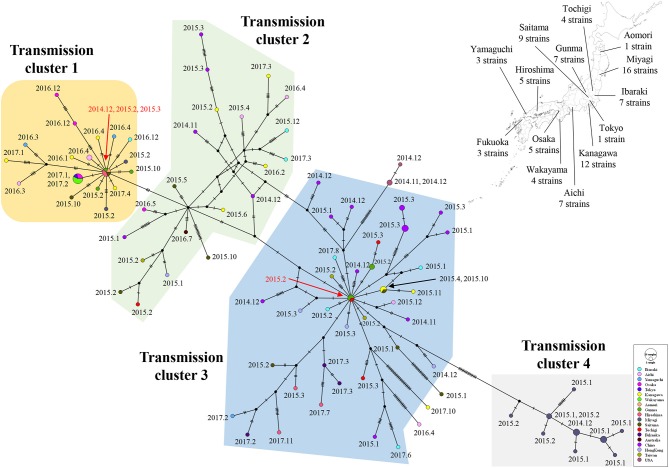
A median joining network for the major epidemic cluster of GII.P17-GII.17 strains based on nucleotide sequences from the *RdRp* to *VP1* (3133 bp). The detected areas for Japanese strains used in the analysis are shown with the number of strains on a Japanese map. The filled circles represent viral haplotypes, including collection years and months for the strains. The sizes of the circles represent the number of strains within the haplotype. Red arrows indicate strains associated with the production of many haplotypes.

Links in circulation of prevalent GII.P17-GII.17 (cluster 1) in Japanese regions and in other countries were analyzed using a median joining network. The strains analyzed in this study were divided into four clusters. Strains belonging to cluster 1 were mainly found from Japan, while those belonging to clusters 2 and 3 were found in various countries including Japan, China, Hong Kong, Taiwan, and Australia. The strains in cluster 4 were detected only in Miyagi prefecture in Japan. Notably, the network contained the key strains that were linked to the many other strains (Figure 7). These results suggest that the GII.P17-GII.17 strains form a broad network and that some strains are associated with the prevalence of the virus.

## Discussion

In this study, we demonstrated the molecular evolution of both the *VP1* and *RdRp* regions of GII.P17-GII.17 since the emergence of the virus. First, a MCMC time-scaled evolutionary phylogenetic tree based on GII.17 *VP1* nucleotide sequences showed that all the present strains grouped into a total of seven clusters (Figure 1). Trees also indicated that the recently emerged GII.P17-GII.17 strains uniquely formed 2 clusters (clusters 1 and 2) based on both the *VP1* and *RdRp* regions. The common ancestor of the *VP1* region of the GII.P17-GII.17 viruses diverged first, followed by divergence of the *RdRp* region after a several-year delay (Figures 1, 2). Previous reports have also suggested a gap in the divergence of GII.P16-GII.2 strains (Mizukoshi et al., 2017; Nagasawa et al., 2018). To resolve this issue, we analyzed the phylogenetic distances of the *VP1* and *RdRp* regions and found that the distance of the *VP1* region was longer than that of *RdRp*, indicating that the genetic diversity of *VP1* in GII.P17-GII.17 is larger than that of *RdRp* (Figure 3). However, the evolutionary rates for the overall cluster in GII.P17-GII.17 were similar between the *VP1* and *RdRp* regions (Table 1). Thus, we also calculated the rates of the strains belonging to the cluster 1, which resulted in reduction of the value in the *RdRp* region, but not in the *VP1* region (data not shown). These results suggest that the differences of divergent times and genetic distances between these regions may be associated with those in the changes of the evolutionary rates. Furthermore, we analyzed the time-scaled MCMC phylogenetic trees by adding 343 sequences of GII.17 for the *VP1*, 133 sequences of GII.P17 for the *RdRp* and data of not only collection years but also months, which produced confidence intervals (95% HPD values) that were smaller in our analyses than in an earlier report (Sang and Yang, 2018). Thus, more precise collection data, as well as the use of a large number of virus strains, may facilitate the construction of more precise MCMC phylogeny.

The evolutionary rates of the *VP1* and *RdRp* regions in the GII.P17-GII.17 were ~2.7 and ~2.3 × 10^−3^ substitutions/site/year, respectively (Table 1). The rate for GII.17 is likely similar to that for GII.2 in the *VP1* region, but lower than that of GII.4 (Bok et al., 2009; Siebenga et al., 2010; Mizukoshi et al., 2017; Motoya et al., 2017). Furthermore, the rate for GII.P17 is likely analogous to that for GII.P16, but lower than that of GII.P4 (Nagasawa et al., 2018; Ozaki et al., 2018). Interestingly, it has been suggested that GII.P4-GII.4 virus prevalence is associated with the rapid evolution of the *VP1* regions with a high ratio of non-synonymous amino acid substitutions to synonymous substitutions (Bull et al., 2010; Parra et al., 2017). Previous reports have also suggested that the rates of non-synonymous amino substitutions in the VP1 protein differ between GII.17 and GII.4, with GII.4 being greater than GII.17 (Mori et al., 2017); therefore, the rates of antigenicity change between GII.17 and GII.4 viruses may be distinct. In contrast, it has been estimated that the number of negative selection sites in GII.P4 *RdRp* are larger than that in GII.P17 (Ozaki et al., 2018), and thus variations in nucleotide substitution in GII.P4 *RdRp* may be restricted compared to GII.P17. However, despite such restrictions, we observed a lower evolutionary rate in GII.P17 than GII.P4. This may be due to differences in replication activities and/or in replication errors of the RdRp protein between GII.P17 and GII.P4. Additional *in vitro* studies may be needed to address these possibilities.

We also assessed the phylodynamics of GII.P17-GII.17 and found that the genome population sizes of GII.P17-GII.17 increased rapidly during 2014–2015 and then decreased rapidly thereafter (Figure 4). These fluctuations may be associated with epidemics of the newly emerged HuNoV and the acquisition of herd immunity. For example, the fluctuations in the phylodynamics of the GII.4 virus could be associated with the emergence of a variant virus with altered antigenicity and the acquisition of herd immunity to the VP1 capsid protein of that virus (Debbink et al., 2013; Lindesmith et al., 2013; Motoya et al., 2017). Thus, it is possible that the phylodynamic fluctuations in GII.P17-GII.17 identified in this study are associated with epidemics in various regions and subsequent acquisition of herd immunity (Chan et al., 2015; Fu et al., 2015; Matsushima et al., 2015; Dang Thanh et al., 2016). These results suggest that continuous phylodynamic analysis may provide early notice of other HuNoV epidemics and repeat prevalence of HuNoV, including GII.17.

We additionally found amino acid substitutions in the conformational epitopes of the VP1 capsid protein of GII.P17-GII.17 (Figure 5 and Table 2). Such substitutions were also identified around the HBGA binding sites. Such evolutionary substitutions may induce both changes in antigenicity and in HBGA binding ability (Tan et al., 2009; Chen et al., 2011; de Rougemont et al., 2011; Lindesmith et al., 2012; Debbink et al., 2013; Jin et al., 2015). Indeed, Lindesmith et al. showed that some amino acid substitutions (corresponding to aa394-aa397 in our alignments) are located adjacent of the HBGA binding sites and result in changes in the antigenicity of the GII.17 capsid protein (Lindesmith et al., 2017). Jin et al. also reported changes to the antigenicities and HBGA binding affinities of GII.17 capsid proteins belonging to different phylogenetic clusters (Jin et al., 2016). Moreover, an amino acid substitution (aa376) located adjacent the HBGA binding sites was identified as a possible positive selection site. Previous reports have suggested that aa375 and aa377 are associated with the HBGA binding ability (He et al., 2017; Koromyslova et al., 2017); however, to the best of our knowledge, there have been no reports regarding possible roles of the aa376 substitution in antigenicity and the HBGA binding capability, which is a subject for possible further study.

We also showed that GII.P17-GII.17 contains amino acid substitutions in residues adjacent to the RdRp active sites and the contact surfaces between RdRp monomers (Figure 6). Ng et al. showed that the amino acids around the active sites regulate viral genome replication (Ng et al., 2008). Moreover, substitutions around the monomer contact surfaces of RdRp affect the stability of the dimer and its RNA binding abilities (Chen et al., 2009). Thus, the similar substitutions found in the present study warrant additional investigation into changes in the properties of the RdRp protein. Previous studies have also revealed key amino acids associated with the efficiency of HuNoV genome replication (Bull et al., 2010; Eden et al., 2011). Of these, the threonine residue at aa33 is phosphorylated by a host factor, Akt, and is involved in producing high replication rates (Eden et al., 2011). This residue is located around the interface between monomers in the RdRp structure. The RdRp protein of GII.P4 contains a threonine residue at the position, while that of GII.P17 contains an asparagine (data not shown). This may suggest that the enzyme activity of RdRp differs between the GII.P4 and GII.P17 genotypes.

We constructed a genome network of the GII.P17-GII.17 strains examined in this study and found four major clusters (Figure 7). Notably, the strains belonging to clusters 1 and 4 were detected exclusively in Japan, while the strains belonging to clusters 2 and 3 were detected from various countries. Moreover, the two haplotype strains found in Japan might give rise to many variant types. However, we could not determine specific amino acid substitutions in the strains belonging to clusters 1 and 3, which contained the haplotype strains (data not shown). Thus, these haplotype strains may exhibit no phenotypes acquiring high infectivity due to the mutations, although the reason for this remains unclear at present. Moreover, the strains belonging to cluster 4 had unique amino acid substitutions in p48, p22, protease, RdRp, and VP2 proteins (data not shown). These viruses were found only in Miyagi prefecture in Japan, during short periods and was never detected in other areas, perhaps because these mutations are deleterious to the propagations of the virus. As a limitation, the results of the present analyses may partially be affected by selection bias introduced in the collection of the strains.

In conclusion, the GII.P17-GII.17 virus has evolved differently to GII.P4-GII.4. However, this virus may have the potential to alter its antigenicity, host-binding capability (i.e., HBGA) and genome replication efficiency. Such changes could recurrently generate variants of GII.17 with the potential to produce pandemics such as those caused by GII.4 variant strains. Thus, additional and continuous evolutionary analyses of this genotype should be needed in the future.

## Data Availability Statement

The datasets generated for this study can be found in the GenBank and the accession numbers are as follow; LC369214-LC369222, LC369224, LC369225, LC369227-LC369249, LC369251-LC369258, LC486737-LC486770.

## Ethics Statement

The studies involving human participants were reviewed and approved by the Research and Ethical Committees for the Use of Human Subjects of the National Institute of Infectious Diseases, Tokyo, Japan (No. 576). Informed consent was obtained from all participants, which was acquired from the subjects or their legally acceptable representatives for sample donation.

## Author Contributions

YM, KK, and HK designed this study. YM conducted all bioinformatic analyses and experiments. YD and MK performed the next-generation sequencing. YM, FM, NSa, YU, YO, TM, HT, NN, NSh, HY, RO-N, RS, RT, FT, TT, KS, HS, KN, JA, and HI collected samples and conducted the Sanger sequencing. YM and HK wrote the manuscript. HS, NO, AR, KK, and HK supervised this study. All authors read and approved the manuscript.

### Conflict of Interest

The authors declare that the research was conducted in the absence of any commercial or financial relationships that could be construed as a potential conflict of interest.

## Footnotes

^1^Available online at: https://norovirus.phiresearchlab.org/

^2^https://www.ncbi.nlm.nih.gov/genbank/

^3^Available online at: http://tree.bio.ed.ac.uk/software/tracer/

^4^Available online at: http://tree.bio.ed.ac.uk/software/figtree/

^5^Available online at: https://blast.ncbi.nlm.nih.gov/Blast.cgi
