# Supplementary material for: Evolutionary Analysis of the VP1 and RNA-Dependent RNA Polymerase Regions of Human Norovirus GII.P17-GII.17 in 2013–2017
Source: Front Microbiol. 2019 Sep 27;10:2189. doi: 10.3389/fmicb.2019.02189 (PMC6777354; doi:10.3389/fmicb.2019.02189)
Supplement: Supplementary file 1 [file Data_Sheet_1.docx]

Supplementary Material

**Evolutionary Analysis of the *VP1* and RNA-dependent RNA Polymerase Regions of Human Norovirus GII.17 in 2013–2017**

**Yuki Matsushima, Fuminori Mizukoshi, Naomi Sakon, Yen Hai Doan, Yo Ueki, Yasutaka Ogawa, Takumi Motoya, Hiroyuki Tsukagoshi, Noriko Nakamura, Naoki Shigemoto, Hideaki Yoshitomi, Reiko Okamoto-Nakagawa, Rieko Suzuki, Rika Tsutsui, Fumio Terasoma, Tomoko Takahashi, Kenji Sadamasu, Hideaki Shimizu, Nobuhiko Okabe, Koo Nagasawa, Jumpei Aso, Haruyuki Ishii, Makoto Kuroda, Akihide Ryo, Kazuhiko Katayama*, Hirokazu Kimura***

*** Correspondence:** Hirokazu Kimura: h-kimura@paz.ac.jp, Kazuhiko Katayama: katayama@lisci.kitasato-u.ac.jp

**Supplementary Figure**

**
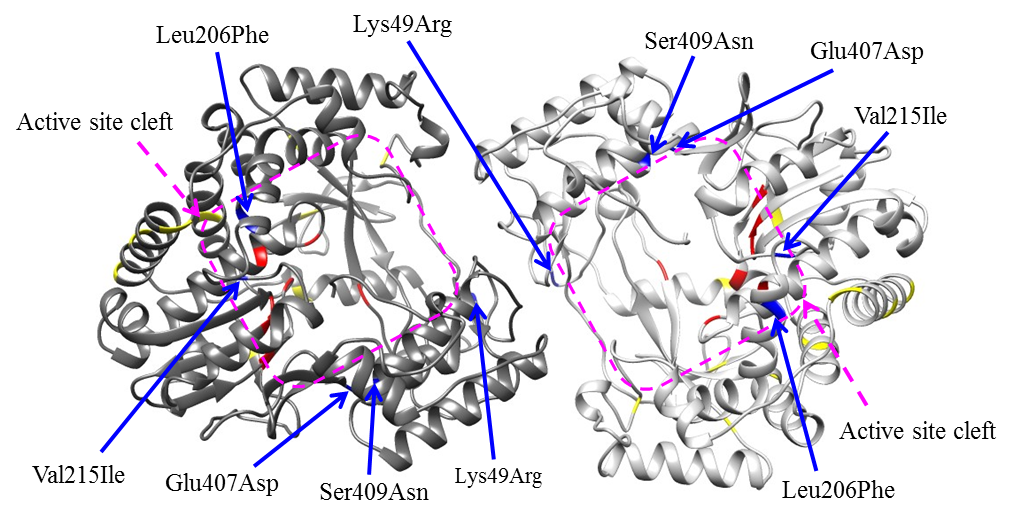
**

**Figure S1.** A structural model of the RdRp protein for representative GII.P3 strain. A homodimer structure of the RdRp of GII.P3 is shown. Each monomeric chain that compose the dimer structure are coloured grey (chain A) and dim grey (chain B). Amino acid substitutions between GII.P17 and GII.P3 that are close to the interface between monomers and to the active site cleft are coloured blue with the positions under the order of alignments, whereas the substitutions distant from these regions are shown in yellow. The residues of active sites for RNA replication are coloured red. The purple quadrilaterals highlight the region of the active site cleft.

**Supplementary Tables**

| Table S1. Strains used in this study | | | | |
| --- | --- | --- | --- | --- |
| Accession numbers | Strains | Collection dates | Genotypes (clusters) | Target genes |
| M87661 | Hu/GI/US/1968/GI.1/Norwalk | 1968 | GI.1 | VP1 |
| U07611 | Hu/GII/US/1971/GII.1/Hawaii_virus | 1971 | GII.1 | VP1 |
| X81879 | Hu/GII/UK/1994/GII.2/Melksham | 1994 | GII.2 | VP1 |
| U02030 | Hu/GII/CA/1991/GII.3/Toronto24 | 1991 | GII.3 | VP1 |
| X76716 | Hu/GII/UK/1993/GII.4_Bristol1993/Bristol | 1993 | GII.4 | VP1 |
| AJ277607 | Hu/GII/UK/1990/GII.5/Hillingdon | 1990 | GII.5 | VP1 |
| JN699035 | Hu/GII/SN/1976/GII.6/S9c | March, 1976 | GII.6 | VP1 |
| JN699042 | Hu/GII/CN/1976/GII.7/HK4 | December, 1976 | GII.7 | VP1 |
| AF195848 | Hu/GII/NE/1998/GII.8/Amsterdam/98-18 | 1998 | GII.8 | VP1 |
| AY038599 | Hu/GII/US/1997/GII.9/VA97207 | 1997 | GII.9 | VP1 |
| AF427118 | Hu/GII/DE/2000/GII.10/Erfurt/546 | 2000 | GII.10 | VP1 |
| AB074893 | Sw/GII/JP/1997/GII.11/Sw918 | 1997 | GII.11 | VP1 |
| KF006267 | Hu/GII/US/2000/GII.12/Texas/E13842 | 2000 | GII.12 | VP1 |
| AY113106 | Hu/GII/US/1998/GII.13/Fayetteville | 1998 | GII.13 | VP1 |
| AY130761 | Hu/GII/US/1999/GII.14/M7 | 1999 | GII.14 | VP1 |
| AY130762 | Hu/GII/US/1999/GII.15/J23 | 1999 | GII.15 | VP1 |
| AY502010 | Hu/GII/US/1999/GII.16/Tiffin | 1999 | GII.16 | VP1 |
| AY823304 | Sw/GII/US/2003/GII.18/OH-QW101 | 2003 | GII.18 | VP1 |
| AY823306 | Sw/GII/US/2003/GII.19/OH-QW170 | 2003 | GII.19 | VP1 |
| EU373815 | Hu/GII/DE/2002/GII.20/Luckenwalde591 | 2002 | GII.20 | VP1 |
| AB542915 | Hu/GII/JP/2005/GII.21/OC05024 | 2005 | GII.21 | VP1 |
| AB083780 | Hu/GII/JP/2002/GII.22/YURI | 2002 | GII.22 | VP1 |

| Accession numbers | Strains | Collection dates | Genotypes  (clusters) | Target genes |
| --- | --- | --- | --- | --- |
| KF429761 | Hu/GI/US/1972/GI.P1/8MoIIIL | June, 1972 | GI.P1 | RdRp |
| JX289822 | Hu/GII/US/1971/GII.P1/Hawaii/7EK | 1971 | GII.P1 | RdRp |
| DQ456824 | Hu/GII/JP/2004/GII.P2/MK04 | 2004 | GII.P2 | RdRp |
| KJ194500 | Hu/GII/NE/1995/GII.P3/Amsterdam/1 | January, 1995 | GII.P3 | RdRp |
| FJ537137 | Hu/GII/US/1987/GII.P4/CHDC4108 | 1987 | GII.P4 | RdRp |
| KJ196288 | Hu/GII/JP/2002/GII.P5/Saitama/T52 | 2002 | GII.P5 | RdRp |
| JX989075 | Hu/GII/CN/2011/GII.P6/Guangzhou/GZ2010-L96 | January, 2011 | GII.P6 | RdRp |
| JX846927 | Hu/GII/US/1984/GII.P7/CHDC4073 | December, 1984 | GII.P7 | RdRp |
| JX846926 | Hu/GII/US/1988/GII.P8/CHDC3936 | September, 1988 | GII.P8 | RdRp |
| HQ392821 | Pi/GII/CN/2009/GII.P11/Ch6 | August, 2009 | GII.P11 | RdRp |
| KJ196294 | Hu/GII/JP/2000/GII.P12/Saitama/KU16 | 2000 | GII.P12 | RdRp |
| KJ196290 | Hu/GII/JP/2007/GII.P15/Sapporo/HK299 | 2007 | GII.P15 | RdRp |
| KJ196286 | Hu/GII/JP/2002/GII.P16/Saitama/T87 | 2002 | GII.P16 | RdRp |
| AY823305 | Sw/GII/US/2003/GII.P18/OH-QW125 | 2003 | GII.P18 | RdRp |
| EU424333 | Hu/GII/DE/2005/GII.P20/Leverkusen267 | 2005 | GII.P20 | RdRp |
| KJ196284 | Hu/GII/JP/2007/GII.P21/Kawasaki/YO284 | 2007 | GII.P21 | RdRp |
| KM036379 | Hu/GII/TW/2012/GII.P22/Taoyuan/12-BB-4 | March, 2012 | GII.P22 | RdRp |
| KF429769 | Hu/GII/US/1975/GII.Pc/SnowMountRS | February, 1975 | GII.Pc | RdRp |
| AB541319 | Hu/GII/JP/2007/GII.Pe/Osaka1 | 2007 | GII.Pe | RdRp |
| KC597144 | Hu/GII/CN/1977/GII.Pg/HK46 | December, 1977 | GII.Pg | RdRp |
| KC576911 | Hu/GII/CAF/1977/GII.Pj/B17 | August, 1977 | GII.Pj | RdRp |
| KJ194507 | Hu/GII/NE/1995/GII.Pm/Amsterdam/3 | January, 1995 | GII.Pm | RdRp |
| KJ196291 | Hu/GII/JP/2011/GII.Pp/Yuzawa/Gira2HS | 2011 | GII.Pp | RdRp |

| Accession numbers | Strains | Collection dates | Genotypes  (clusters) | Target genes |
| --- | --- | --- | --- | --- |
| KY905332 | Hu/GII/AU/2015/GII.P17-GII.17/NSW543Q | June, 2015 | GII.17 (cluster 2) | VP1, RdRp |
| KP902565 | Hu/GII/HKG/2014/GII.17/CUHK-NS-360 | August, 2014 | GII.17 (cluster 2) | VP1 |
| KX171416 | Hu/GII/CA/2015/GII.17/AlbertaSG005 | March, 2015 | GII.17 (cluster 2) | VP1 |
| KU557788 | Hu/GII/CN/2013/GII.P17-GII.17/2238/GD-JM | August, 2013 | GII.17 (cluster 2) | VP1, RdRp |
| LC043168 | Hu/GII/JP/2013/GII.P17-GII.17/Saitama5309 | July, 2013 | GII.17 (cluster 2) | VP1, RdRp |
| AB983218 | Hu/GII/JP/2014/GII.P17-GII.17/Kawasaki323 | March, 2014 | GII.17 (cluster 2) | VP1, RdRp |
| LC043139 | Hu/GII/JP/2014/GII.P17-GII.17/Nagano7-1 | August, 2014 | GII.17 (cluster 2) | VP1, RdRp |
| LC043305 | Hu/GII/JP/2014/GII.P17-GII.17/Nagano8-1 | August, 2014 | GII.17 (cluster 2) | VP1, RdRp |
| LC043167 | Hu/GII/JP/2013/GII.P17-GII.17/Saitama5203 | April, 2013 | GII.17 (cluster 2) | VP1, RdRp |
| KJ156329 | Hu/GII/TW/2013/GII.P17-GII.17/13-BH-1 | October, 2013 | GII.17 (cluster 2) | VP1 |
| KP902563 | Hu/GII/HKG/2014/GII.17/CUHK-NS-258 | March, 2014 | GII.17 (cluster 2) | VP1 |
| KP902564 | Hu/GII/HKG/2014/GII.17/CUHK-NS-276 | April, 2014 | GII.17 (cluster 2) | VP1 |
| LC369251 | Hu/GII/JP/2015/GII.P17-GII.17/SA-164 | August, 2015 | GII.17 (cluster 2) | VP1, RdRp |
| KX171412 | Hu/GII/CA/2014/GII.17/AlbertaEI421 | November, 2014 | GII.17 (cluster 2) | VP1 |
| KX171413 | Hu/GII/CA/2014/GII.17/AlbertaEI487 | December, 2014 | GII.17 (cluster 2) | VP1 |
| KT285173 | Hu/GII/AU/2014/GII.P17-GII.17/NSW6016 | May, 2014 | GII.17 (cluster 2) | VP1 |
| KX171417 | Hu/GII/CA/2014/GII.17/AlbertaSG001 | December, 2014 | GII.17 (cluster 2) | VP1 |
| KP676383 | Hu/GII/CN/2013/GII.P17-GII.17/Nanjing010141 | June, 2013 | GII.17 (cluster 2) | RdRp |
| KU557789 | Hu/GII/CN/2013/GII.P17-GII.17/GD-FS/1421 | September, 2013 | GII.17 (cluster 2) | RdRp |
| LC486738 | Hu/GII/JP/2014/GII.P17-GII.17/IB-N14-342 | August, 2014 | GII.17 (cluster 2) | VP1, RdRp |
| LC486737 | Hu/GII/JP/2013/GII.P17-GII.17/IB-N13-89 | June, 2013 | GII.17 (cluster 2) | VP1, RdRp |
| LC101820 | Hu/GII/JP/2015/GII.17/MIY2 | February, 2015 | GII.17 (cluster 1) | VP1 |
| KU561224 | Hu/GII/HKG/2014/GII.17/CUHK-NS-471 | December, 2014 | GII.17 (cluster 1) | VP1 |
| KU561225 | Hu/GII/HKG/2014/GII.17/CUHK-NS-477 | December, 2014 | GII.17 (cluster 1) | VP1 |
| KU557800 | Hu/GII/CN/2015/GII.17/GD-GZ/12-0119 | January, 2015 | GII.17 (cluster 1) | VP1 |
| KU557801 | Hu/GII/CN/2013/GII.17/GD-GZ/1209 | December, 2013 | GII.17 (cluster 1) | VP1 |
| KU557833 | Hu/GII/CN/2014/GII.17/GD-JM/21-1712 | December, 2014 | GII.17 (cluster 1) | VP1 |
| KU557834 | Hu/GII/CN/2014/GII.17/GD-JM/21-1713 | December, 2014 | GII.17 (cluster 1) | VP1 |
| KU557835 | Hu/GII/CN/2014/GII.17/GD-JM/21-1714 | December, 2014 | GII.17 (cluster 1) | VP1 |
| KT780401 | Hu/GII/HKG/2015/GII.P17-GII.17/CUHK-NS-517 | January, 2015 | GII.17 (cluster 1) | VP1, RdRp |
| KR052019 | Hu/GII/TW/2015/GII.P17-GII.17/Taichung/15-AD-2 | February, 2015 | GII.17 (cluster 1) | VP1, RdRp |
| LC258403 | Hu/GII/JP/2017/GII.P17-GII.17/Tokyo330021 | February, 2017 | GII.17 (cluster 1) | VP1, RdRp |
| KU557784 | Hu/GII/CN/2015/GII.P17-GII.17/GD-JM/35-0585 | March, 2015 | GII.17 (cluster 1) | VP1, RdRp |
| LC037415 | Hu/GII/JP/2015/GII.P17-GII.17/Kawasaki308 | February, 2015 | GII.17 (cluster 1) | VP1, RdRp |
| KU557783 | Hu/GII/CN/2015/GII.P17-GII.17/GD-JM/35-0584 | March, 2015 | GII.17 (cluster 1) | VP1, RdRp |
| KT970374 | Hu/GII/CN/2015/GII.P17-GII.17/Guangzhou/GZ2015-L339 | January, 2015 | GII.17 (cluster 1) | VP1, RdRp |

Collected samples in this study are shown by red characters.

| Accession numbers | Strains | Collection dates | Genotypes  (clusters) | Target genes |
| --- | --- | --- | --- | --- |
| KP902573 | Hu/GII/HKG/2014/GII.17/CUHK-NS-483 | December, 2014 | GII.17 (cluster 1) | VP1 |
| KU557787 | Hu/GII/CN/2014/GII.P17-GII.17/GD-JM/18-1712 | December, 2014 | GII.17 (cluster 1) | VP1, RdRp |
| KY905330 | Hu/GII/AU/2016/GII.P17-GII.17/NSW9428 | July, 2016 | GII.17 (cluster 1) | VP1, RdRp |
| LC148856 | Hu/GII/JP/2016/GII.17/Osaka15-493 | March, 2016 | GII.17 (cluster 1) | VP1 |
| LC148852 | Hu/GII/JP/2016/GII.17/Osaka15-377 | January, 2016 | GII.17 (cluster 1) | VP1 |
| LC148849 | Hu/GII/JP/2015/GII.17/Osaka14-508 | March, 2015 | GII.17 (cluster 1) | VP1 |
| KR052022 | Hu/GII/TW/2015/GII.P17-GII.17/Yunlin/15-R-4 | January, 2015 | GII.17 (cluster 1) | VP1 |
| KU561233 | Hu/GII/HKG/2015/GII.17/CUHK-NS-541 | January, 2015 | GII.17 (cluster 1) | VP1 |
| KU557862 | Hu/GII/CN/2015/GII.17/GD-JM/35-0586 | March, 2015 | GII.17 (cluster 1) | VP1 |
| KU557864 | Hu/GII/CN/2015/GII.17/GD-ZQ/37-0719 | April, 2015 | GII.17 (cluster 1) | VP1 |
| KU557865 | Hu/GII/CN/2015/GII.17/GD-ZQ/37-0720 | April, 2015 | GII.17 (cluster 1) | VP1 |
| LC148844 | Hu/GII/JP/2015/GII.17/Osaka14-273 | January, 2015 | GII.17 (cluster 1) | VP1 |
| LC148845 | Hu/GII/JP/2015/GII.17/Osaka14-283 | January, 2015 | GII.17 (cluster 1) | VP1 |
| LC148846 | Hu/GII/JP/2015/GII.17/Osaka14-332 | January, 2015 | GII.17 (cluster 1) | VP1 |
| LC148847 | Hu/GII/JP/2015/GII.17/Osaka14-346 | January, 2015 | GII.17 (cluster 1) | VP1 |
| KU557863 | Hu/GII/CN/2015/GII.17/GD-ZQ/37-0718 | April, 2015 | GII.17 (cluster 1) | VP1 |
| KP902569 | Hu/GII/HKG/2014/GII.17/CUHK-NS-456 | November, 2014 | GII.17 (cluster 1) | VP1 |
| LC148848 | Hu/GII/JP/2015/GII.17/Osaka14-394 | February, 2015 | GII.17 (cluster 1) | VP1 |
| KU561231 | Hu/GII/HKG/2015/GII.17/CUHK-NS-535 | January, 2015 | GII.17 (cluster 1) | VP1 |
| KP864102 | Hu/GII/CN/2015/GII.17/Shanghai/152642 | January, 2015 | GII.17 (cluster 1) | VP1 |
| LC148855 | Hu/GII/JP/2016/GII.17/Osaka15-479 | March, 2016 | GII.17 (cluster 1) | VP1 |
| LC486761 | Hu/GII/JP/2015/GII.P17-GII.17/KS-19-1 | June, 2015 | GII.17 (cluster 1) | VP1, RdRp |
| LC318755 | Hu/GII/JP/2017/GII.P17-GII.17/Wakayama_K14 | January, 2017 | GII.17 (cluster 1) | VP1, RdRp |
| LC318757 | Hu/GII/JP/2017/GII.P17-GII.17/Wakayama_J2 | January, 2017 | GII.17 (cluster 1) | VP1, RdRp |
| LC318756 | Hu/GII/JP/2017/GII.P17-GII.17/Wakayama_K3 | January, 2017 | GII.17 (cluster 1) | VP1, RdRp |
| LC318758 | Hu/GII/JP/2017/GII.P17-GII.17/Wakayama_J7 | January, 2017 | GII.17 (cluster 1) | VP1, RdRp |
| LC369214 | Hu/GII/JP/2014/GII.P17-GII.17/AO-08 | December, 2014 | GII.17 (cluster 1) | VP1, RdRp |
| LC369215 | Hu/GII/JP/2015/GII.P17-GII.17/GU-55 | February, 2015 | GII.17 (cluster 1) | VP1, RdRp |
| LC369216 | Hu/GII/JP/2015/GII.P17-GII.17/GU-56 | February, 2015 | GII.17 (cluster 1) | VP1, RdRp |
| LC369221 | Hu/GII/JP/2015/GII.P17-GII.17/GU-69 | October, 2015 | GII.17 (cluster 1) | VP1, RdRp |
| LC369222 | Hu/GII/JP/2015/GII.P17-GII.17/HI-78 | March, 2015 | GII.17 (cluster 1) | VP1, RdRp |
| LC369224 | Hu/GII/JP/2015/GII.P17-GII.17/HI-80 | March, 2015 | GII.17 (cluster 1) | VP1, RdRp |
| LC369241 | Hu/GII/JP/2015/GII.P17-GII.17/MI-92 | February, 2015 | GII.17 (cluster 1) | VP1, RdRp |
| LC369242 | Hu/GII/JP/2015/GII.P17-GII.17/MI-93 | February, 2015 | GII.17 (cluster 1) | VP1, RdRp |
| LC369244 | Hu/GII/JP/2015/GII.P17-GII.17/SA-116 | May, 2015 | GII.17 (cluster 1) | VP1, RdRp |
| LC369248 | Hu/GII/JP/2015/GII.P17-GII.17/SA-149 | February, 2015 | GII.17 (cluster 1) | VP1, RdRp |

Collected samples in this study are shown by red characters.

| Accession numbers | Strains | Collection dates | Genotypes  (clusters) | Target genes |
| --- | --- | --- | --- | --- |
| LC369249 | Hu/GII/JP/2015/GII.P17-GII.17/SA-150 | February, 2015 | GII.17 (cluster 1) | VP1, RdRp |
| LC369253 | Hu/GII/JP/2015/GII.P17-GII.17/SA-172 | October, 2015 | GII.17 (cluster 1) | VP1, RdRp |
| LC369254 | Hu/GII/JP/2015/GII.P17-GII.17/TO-144 | February, 2015 | GII.17 (cluster 1) | VP1, RdRp |
| LC486764 | Hu/GII/JP/2016/GII.P17-GII.17/KS-19-4 | January, 2016 | GII.17 (cluster 1) | VP1, RdRp |
| LC486765 | Hu/GII/JP/2016/GII.P17-GII.17/KS-19-5 | February, 2016 | GII.17 (cluster 1) | VP1, RdRp |
| LC486766 | Hu/GII/JP/2016/GII.P17-GII.17/KS-19-6 | April, 2016 | GII.17 (cluster 1) | VP1, RdRp |
| LC486768 | Hu/GII/JP/2017/GII.P17-GII.17/KS-19-8 | January, 2017 | GII.17 (cluster 1) | VP1, RdRp |
| LC486767 | Hu/GII/JP/2017/GII.P17-GII.17/KS-19-7 | April, 2017 | GII.17 (cluster 1) | VP1, RdRp |
| LC318745 | Hu/GII/JP/2016/GII.P17-GII.17/OsakaFB64 | May, 2016 | GII.17 (cluster 1) | VP1, RdRp |
| LC318747 | Hu/GII/JP/2016/GII.P17-GII.17/OsakaFB311 | December, 2016 | GII.17 (cluster 1) | VP1, RdRp |
| LC318753 | Hu/GII/JP/2017/GII.P17-GII.17/OsakaFB433 | February, 2017 | GII.17 (cluster 1) | VP1, RdRp |
| LC318754 | Hu/GII/JP/2017/GII.P17-GII.17/OsakaFB434 | February, 2017 | GII.17 (cluster 1) | VP1, RdRp |
| LC148851 | Hu/GII/JP/2015/GII.17/Osaka15-208 | August, 2015 | GII.17 (cluster 1) | VP1 |
| KX168442 | Hu/GII/HKG/2016/GII.17/CUHK-NS-882 | January, 2016 | GII.17 (cluster 1) | VP1 |
| KU557872 | Hu/GII/CN/2015/GII.17/GD-GZ/40-1012 | April, 2015 | GII.17 (cluster 1) | VP1 |
| KU557873 | Hu/GII/CN/2015/GII.17/GD-GZ/40-1013 | April, 2015 | GII.17 (cluster 1) | VP1 |
| KU557874 | Hu/GII/CN/2015/GII.17/GD-GZ/40-1014 | April, 2015 | GII.17 (cluster 1) | VP1 |
| KU557850 | Hu/GII/CN/2015/GII.17/GD-JY/28-0454 | February, 2015 | GII.17 (cluster 1) | VP1 |
| KU557851 | Hu/GII/CN/2015/GII.17/GD-JY/28-0455 | February, 2015 | GII.17 (cluster 1) | VP1 |
| KU557852 | Hu/GII/CN/2015/GII.17/GD-JY/28-0457 | February, 2015 | GII.17 (cluster 1) | VP1 |
| KP902583 | Hu/GII/HKG/2015/GII.17/CUHK-NS-520 | January, 2015 | GII.17 (cluster 1) | VP1 |
| KU557857 | Hu/GII/CN/2015/GII.17/GD-DG/30-0501 | March, 2015 | GII.17 (cluster 1) | VP1 |
| KU557858 | Hu/GII/CN/2015/GII.17/GD-DG/30-0502 | March, 2015 | GII.17 (cluster 1) | VP1 |
| KU557866 | Hu/GII/CN/2015/GII.17/GD-GZ/38-0965 | March, 2015 | GII.17 (cluster 1) | VP1 |
| KU557867 | Hu/GII/CN/2015/GII.17/GD-GZ/38-0966 | March, 2015 | GII.17 (cluster 1) | VP1 |
| KU557868 | Hu/GII/CN/2015/GII.17/GD-GZ/38-0967 | March, 2015 | GII.17 (cluster 1) | VP1 |
| KX168438 | Hu/GII/HKG/2015/GII.17/CUHK-NS-861 | December, 2015 | GII.17 (cluster 1) | VP1 |
| KX168446 | Hu/GII/HKG/2016/GII.17/CUHK-NS-901 | February, 2016 | GII.17 (cluster 1) | VP1 |
| KX168447 | Hu/GII/HKG/2016/GII.17/CUHK-NS-907 | February, 2016 | GII.17 (cluster 1) | VP1 |
| KX168450 | Hu/GII/HKG/2016/GII.17/CUHK-NS-920 | March, 2016 | GII.17 (cluster 1) | VP1 |
| KX168454 | Hu/GII/HKG/2016/GII.17/CUHK-NS-935 | March, 2016 | GII.17 (cluster 1) | VP1 |
| KX168455 | Hu/GII/HKG/2016/GII.17/CUHK-NS-936 | March, 2016 | GII.17 (cluster 1) | VP1 |
| KX168443 | Hu/GII/HKG/2016/GII.17/CUHK-NS-892 | February, 2016 | GII.17 (cluster 1) | VP1 |
| KX168445 | Hu/GII/HKG/2016/GII.17/CUHK-NS-899 | February, 2016 | GII.17 (cluster 1) | VP1 |
| KX168453 | Hu/GII/HKG/2016/GII.17/CUHK-NS-930 | March, 2016 | GII.17 (cluster 1) | VP1 |
| KU561245 | Hu/GII/HKG/2015/GII.17/CUHK-NS-635 | March, 2015 | GII.17 (cluster 1) | VP1 |

Collected samples in this study are shown by red characters.

| Accession numbers | Strains | Collection dates | Genotypes  (clusters) | Target genes |
| --- | --- | --- | --- | --- |
| KX168439 | Hu/GII/HKG/2016/GII.17/CUHK-NS-863 | January, 2016 | GII.17 (cluster 1) | VP1 |
| KX168440 | Hu/GII/HKG/2016/GII.17/CUHK-NS-866 | January, 2016 | GII.17 (cluster 1) | VP1 |
| KT591501 | Hu/GII/SI/2015/GII.17/Ljubljana1662 | June, 2015 | GII.17 (cluster 1) | VP1 |
| KU561226 | Hu/GII/HKG/2014/GII.17/CUHK-NS-481 | December, 2014 | GII.17 (cluster 1) | VP1 |
| KU561236 | Hu/GII/HKG/2015/GII.17/CUHK-NS-548 | January, 2015 | GII.17 (cluster 1) | VP1 |
| KU557848 | Hu/GII/CN/2014/GII.17/GD-GZ/27-2055 | December, 2014 | GII.17 (cluster 1) | VP1 |
| KU557849 | Hu/GII/CN/2014/GII.17/GD-GZ/27-2058 | December, 2014 | GII.17 (cluster 1) | VP1 |
| KR020503 | Hu/GII/CN/2014/GII.P17-GII.17/Guangzhou/41621 | December, 2014 | GII.17 (cluster 1) | VP1, RdRp |
| KP902575 | Hu/GII/HKG/2014/GII.17/CUHK-NS-493 | December, 2014 | GII.17 (cluster 1) | VP1 |
| KU561228 | Hu/GII/HKG/2014/GII.17/CUHK-NS-488 | December, 2014 | GII.17 (cluster 1) | VP1 |
| KU561241 | Hu/GII/HKG/2015/GII.17/CUHK-NS-595 | February, 2015 | GII.17 (cluster 1) | VP1 |
| KU561234 | Hu/GII/HKG/2015/GII.17/CUHK-NS-543 | January, 2015 | GII.17 (cluster 1) | VP1 |
| KU561244 | Hu/GII/HKG/2015/GII.17/CUHK-NS-622 | March, 2015 | GII.17 (cluster 1) | VP1 |
| KU557815 | Hu/GII/CN/2014/GII.17/GD-DG/16-1669 | December, 2014 | GII.17 (cluster 1) | VP1 |
| KU557817 | Hu/GII/CN/2014/GII.17/GD-DG/16-1671 | December, 2014 | GII.17 (cluster 1) | VP1 |
| KU557816 | Hu/GII/CN/2014/GII.17/GD-DG/16-1670 | December, 2014 | GII.17 (cluster 1) | VP1 |
| KP902587 | Hu/GII/HKG/2015/GII.17/CUHK-NS-556 | January, 2015 | GII.17 (cluster 1) | VP1 |
| KP698928 | Hu/GII/HKG/2014/GII.17/CUHK-NS-491 | December, 2014 | GII.17 (cluster 1) | VP1 |
| KU557799 | Hu/GII/CN/2014/GII.17/GD-DG/1184 | August, 2014 | GII.17 (cluster 1) | VP1 |
| KU557855 | Hu/GII/CN/2015/GII.17/GD-HY/3-0014 | January, 2015 | GII.17 (cluster 1) | VP1 |
| KU557856 | Hu/GII/CN/2015/GII.17/GD-HY/3-0016 | January, 2015 | GII.17 (cluster 1) | VP1 |
| KP864104 | Hu/GII/CN/2014/GII.17/Shanghai/142661 | December, 2014 | GII.17 (cluster 1) | VP1 |
| KT149174 | Hu/GII/CN/2015/GII.17/Guangzhou/GZ2015-L340 | January, 2015 | GII.17 (cluster 1) | VP1 |
| KU561248 | Hu/GII/HKG/2015/GII.P17-GII.17/CUHK-NS-613 | March, 2015 | GII.17 (cluster 1) | VP1, RdRp |
| KU561249 | Hu/GII/HKG/2015/GII.P17-GII.17/CUHK-NS-616 | March, 2015 | GII.17 (cluster 1) | VP1, RdRp |
| KR052021 | Hu/GII/TW/2015/GII.P17-GII.17/Hsinchu/15-AP-1 | February, 2015 | GII.17 (cluster 1) | VP1, RdRp |
| KX168441 | Hu/GII/HKG/2016/GII.17/CUHK-NS-880 | January, 2016 | GII.17 (cluster 1) | VP1 |
| KU557802 | Hu/GII/CN/2014/GII.17/GD-JM/12-1515 | November, 2014 | GII.17 (cluster 1) | VP1 |
| KU557803 | Hu/GII/CN/2014/GII.17/GD-JM/12-1516 | November, 2014 | GII.17 (cluster 1) | VP1 |
| KU557822 | Hu/GII/CN/2014/GII.17/GD-JM/18-1680 | December, 2014 | GII.17 (cluster 1) | VP1 |
| KU557823 | Hu/GII/CN/2014/GII.17/GD-JM/18-1681 | December, 2014 | GII.17 (cluster 1) | VP1 |
| KU557824 | Hu/GII/CN/2014/GII.17/GD-JM/18-1682 | December, 2014 | GII.17 (cluster 1) | VP1 |
| KP902571 | Hu/GII/HKG/2014/GII.17/CUHK-NS-480 | December, 2014 | GII.17 (cluster 1) | VP1 |
| LC148850 | Hu/GII/JP/2015/GII.17/Osaka15-157 | July, 2015 | GII.17 (cluster 1) | VP1 |
| KP902568 | Hu/GII/HKG/2014/GII.17/CUHK-NS-455 | November, 2014 | GII.17 (cluster 1) | VP1 |
| KP902567 | Hu/GII/HKG/2014/GII.17/CUHK-NS-438 | November, 2014 | GII.17 (cluster 1) | VP1 |

| Accession numbers | Strains | Collection dates | Genotypes  (clusters) | Target genes |
| --- | --- | --- | --- | --- |
| KU561229 | Hu/GII/HKG/2014/GII.17/CUHK-NS-498 | December, 2014 | GII.17 (cluster 1) | VP1 |
| KU561232 | Hu/GII/HKG/2015/GII.17/CUHK-NS-536 | January, 2015 | GII.17 (cluster 1) | VP1 |
| KU557811 | Hu/GII/CN/2014/GII.17/GD-MM/1549 | November, 2014 | GII.17 (cluster 1) | VP1 |
| KU557785 | Hu/GII/CN/2014/GII.P17-GII.17/GD-MM/1085 | November, 2014 | GII.17 (cluster 1) | VP1, RdRp |
| KP902580 | Hu/GII/HKG/2015/GII.17/CUHK-NS-512 | January, 2015 | GII.17 (cluster 1) | VP1 |
| KU557818 | Hu/GII/CN/2015/GII.17/GD-JM/17-0151 | January, 2015 | GII.17 (cluster 1) | VP1 |
| KU557819 | Hu/GII/CN/2015/GII.17/GD-JM/17-0152 | January, 2015 | GII.17 (cluster 1) | VP1 |
| KU557820 | Hu/GII/CN/2015/GII.17/GD-JM/17-0153 | January, 2015 | GII.17 (cluster 1) | VP1 |
| KU557830 | Hu/GII/CN/2015/GII.17/GD-GZ/21-0193 | January, 2015 | GII.17 (cluster 1) | VP1 |
| KU557831 | Hu/GII/CN/2015/GII.17/GD-GZ/21-0194 | January, 2015 | GII.17 (cluster 1) | VP1 |
| KU557832 | Hu/GII/CN/2015/GII.17/GD-GZ/21-0195 | January, 2015 | GII.17 (cluster 1) | VP1 |
| KU557836 | Hu/GII/CN/2015/GII.17/GD-GZ/22-0203 | February, 2015 | GII.17 (cluster 1) | VP1 |
| KU557837 | Hu/GII/CN/2015/GII.17/GD-GZ/22-0204 | February, 2015 | GII.17 (cluster 1) | VP1 |
| KU557838 | Hu/GII/CN/2015/GII.17/GD-GZ/22-0205 | February, 2015 | GII.17 (cluster 1) | VP1 |
| KP902585 | Hu/GII/HKG/2015/GII.17/CUHK-NS-528 | January, 2015 | GII.17 (cluster 1) | VP1 |
| KU557882 | Hu/GII/CN/2015/GII.17/GD-ZH/5-0033 | January, 2015 | GII.17 (cluster 1) | VP1 |
| KU557841 | Hu/GII/CN/2015/GII.17/GD-DG/24-0221 | January, 2015 | GII.17 (cluster 1) | VP1 |
| KU557842 | Hu/GII/CN/2015/GII.17/GD-DG/24-0222 | January, 2015 | GII.17 (cluster 1) | VP1 |
| KU557821 | Hu/GII/CN/2015/GII.17/GD-MM/18-0158 | January, 2015 | GII.17 (cluster 1) | VP1 |
| KT970372 | Hu/GII/CN/2015/GII.P17-GII.17/Guangzhou/GZ2015-L325 | January, 2015 | GII.17 (cluster 1) | VP1, RdRp |
| KT970369  KT149168 | Hu/GII/CN/2014/GII.P17-GII.17/Guangzhou/GZ2014-L311 | December, 2014 | GII.17 (cluster 1) | VP1, RdRp |
| LC148853 | Hu/GII/JP/2016/GII.17/Osaka15-399 | January, 2016 | GII.17 (cluster 1) | VP1 |
| KX171414 | Hu/GII/CA/2015/GII.17/AlbertaEI331 | August, 2015 | GII.17 (cluster 1) | VP1 |
| KU557840 | Hu/GII/CN/2015/GII.17/GD-DG/23-0216 | January, 2015 | GII.17 (cluster 1) | VP1 |
| KU557843 | Hu/GII/CN/2015/GII.17/GD-ZH/25-0224 | January, 2015 | GII.17 (cluster 1) | VP1 |
| KU557844 | Hu/GII/CN/2015/GII.17/GD-ZH/25-0225 | January, 2015 | GII.17 (cluster 1) | VP1 |
| KU557845 | Hu/GII/CN/2015/GII.17/GD-DG/25-0227 | January, 2015 | GII.17 (cluster 1) | VP1 |
| KU557889 | Hu/GII/CN/2015/GII.17/GD-ZH/8-0089 | January, 2015 | GII.17 (cluster 1) | VP1 |
| KU557890 | Hu/GII/CN/2015/GII.17/GD-ZH/8-0090 | January, 2015 | GII.17 (cluster 1) | VP1 |
| KU557891 | Hu/GII/CN/2015/GII.17/GD-ZH/8-0091 | January, 2015 | GII.17 (cluster 1) | VP1 |
| KU557885 | Hu/GII/CN/2014/GII.17/GD-ZH/6-0046 | December, 2014 | GII.17 (cluster 1) | VP1 |
| KU557825 | Hu/GII/CN/2015/GII.17/GD-GZ/20-0181 | January, 2015 | GII.17 (cluster 1) | VP1 |
| KU557826 | Hu/GII/CN/2015/GII.17/GD-GZ/20-0183 | January, 2015 | GII.17 (cluster 1) | VP1 |
| KP902590 | Hu/GII/HKG/2015/GII.17/CUHK-NS-575 | January, 2015 | GII.17 (cluster 1) | VP1 |
| KU557804 | Hu/GII/CN/2015/GII.17/GD-JM/14-0134 | January, 2015 | GII.17 (cluster 1) | VP1 |

| Accession numbers | Strains | Collection dates | Genotypes  (clusters) | Target genes |
| --- | --- | --- | --- | --- |
| KU557805 | Hu/GII/CN/2015/GII.17/GD-JM/14-0135 | January, 2015 | GII.17 (cluster 1) | VP1 |
| KU557846 | Hu/GII/CN/2014/GII.17/GD-GZ/26-2044 | December, 2014 | GII.17 (cluster 1) | VP1 |
| KU557847 | Hu/GII/CN/2014/GII.17/GD-GZ/26-2047 | December, 2014 | GII.17 (cluster 1) | VP1 |
| KU557809 | Hu/GII/CN/2014/GII.17/GD-QY/15-1651 | December, 2014 | GII.17 (cluster 1) | VP1 |
| KP902579 | Hu/GII/HKG/2015/GII.17/CUHK-NS-506 | January, 2015 | GII.17 (cluster 1) | VP1 |
| KU561247 | Hu/GII/HKG/2015/GII.17/CUHK-NS-680 | June, 2015 | GII.17 (cluster 1) | VP1 |
| KY069115 | Hu/GII/HKG/2016/GII.17/CUHK-NS-1011 | August, 2016 | GII.17 (cluster 1) | VP1 |
| KP902578 | Hu/GII/HKG/2014/GII.17/CUHK-NS-503 | December, 2014 | GII.17 (cluster 1) | VP1 |
| KU561240 | Hu/GII/HKG/2015/GII.17/CUHK-NS-594 | February, 2015 | GII.17 (cluster 1) | VP1 |
| KT970370 | Hu/GII/CN/2014/GII.P17-GII.17/Guangzhou/GZ2014-L313 | December, 2014 | GII.17 (cluster 1) | VP1, RdRp |
| KP902584 | Hu/GII/HKG/2015/GII.17/CUHK-NS-521 | January, 2015 | GII.17 (cluster 1) | VP1 |
| KU557806 | Hu/GII/CN/2014/GII.17/GD-SZ/14-1533 | November, 2014 | GII.17 (cluster 1) | VP1 |
| KU557892 | Hu/GII/CN/2015/GII.17/GD-ZQ/9-0097 | January, 2015 | GII.17 (cluster 1) | VP1 |
| KU557893 | Hu/GII/CN/2015/GII.17/GD-ZQ/9-0098 | January, 2015 | GII.17 (cluster 1) | VP1 |
| KT970373 | Hu/GII/CN/2015/GII.P17-GII.17/Guangzhou/GZ2015-L337 | January, 2015 | GII.17 (cluster 1) | VP1, RdRp |
| KT970377 | Hu/GII/CN/2015/GII.P17-GII.17/Guangzhou/GZ2015-L362 | March, 2015 | GII.17 (cluster 1) | VP1, RdRp |
| KP902581 | Hu/GII/HKG/2015/GII.17/CUHK-NS-514 | January, 2015 | GII.17 (cluster 1) | VP1 |
| KP698931 | Hu/GII/HKG/2015/GII.17/CUHK-NS-513 | January, 2015 | GII.17 (cluster 1) | VP1 |
| KU561243 | Hu/GII/HKG/2015/GII.17/CUHK-NS-614 | March, 2015 | GII.17 (cluster 1) | VP1 |
| KX168451 | Hu/GII/HKG/2016/GII.17/CUHK-NS-922 | March, 2016 | GII.17 (cluster 1) | VP1 |
| KU557853 | Hu/GII/CN/2015/GII.17/GD-ZQ/29-0499 | March, 2015 | GII.17 (cluster 1) | VP1 |
| KU557854 | Hu/GII/CN/2015/GII.17/GD-ZQ/29-0500 | March, 2015 | GII.17 (cluster 1) | VP1 |
| KP998539 | Hu/GII/HKG/2014/GII.P17-GII.17/CUHK-NS-463 | December, 2014 | GII.17 (cluster 1) | VP1, RdRp |
| KU557812 | Hu/GII/CN/2015/GII.17/GD-JM/16-0148 | January, 2015 | GII.17 (cluster 1) | VP1 |
| KU557813 | Hu/GII/CN/2015/GII.17/GD-JM/16-0149 | January, 2015 | GII.17 (cluster 1) | VP1 |
| KU557814 | Hu/GII/CN/2015/GII.17/GD-JM/16-0150 | January, 2015 | GII.17 (cluster 1) | VP1 |
| KT992785 | Hu/GII/CN/2015/GII.P17-GII.17/Nanyang/HN01 | March, 2015 | GII.17 (cluster 1) | VP1, RdRp |
| KT992788 | Hu/GII/CN/2015/GII.P17-GII.17/Nanyang/HN04 | March, 2015 | GII.17 (cluster 1) | VP1, RdRp |
| KT992786 | Hu/GII/CN/2015/GII.P17-GII.17/Nanyang/HN02 | March, 2015 | GII.17 (cluster 1) | VP1 |
| KT992787 | Hu/GII/CN/2015/GII.P17-GII.17/Nanyang/HN03 | March, 2015 | GII.17 (cluster 1) | VP1, RdRp |
| KT992789 | Hu/GII/CN/2015/GII.P17-GII.17/Nanyang/HN05 | March, 2015 | GII.17 (cluster 1) | VP1, RdRp |
| KT992790 | Hu/GII/CN/2015/GII.P17-GII.17/Nanyang/Hnkaohao | March, 2015 | GII.17 (cluster 1) | VP1, RdRp |
| KY069114 | Hu/GII/HKG/2014/GII.17/CUHK-NS-467 | December, 2014 | GII.17 (cluster 1) | VP1 |
| KT149170 | Hu/GII/CN/2015/GII.17/Guangzhou/GZ2015-L324 | January, 2015 | GII.17 (cluster 1) | VP1 |
| KX134671 | Hu/GII/SI/2016/GII.17/Ljubljana535 | March, 2016 | GII.17 (cluster 1) | VP1 |
| KX424646 | Hu/GII/NL/2015/GII.P17-GII.17/Veldhoven219 | February, 2015 | GII.17 (cluster 1) | VP1 |

| Accession numbers | Strains | Collection dates | Genotypes  (clusters) | Target genes |
| --- | --- | --- | --- | --- |
| KX168449 | Hu/GII/HKG/2016/GII.17/CUHK-NS-917 | March, 2016 | GII.17 (cluster 1) | VP1 |
| KX420892 | Hu/GII/CA/2015/GII.17/ABEI446 | December, 2015 | GII.17 (cluster 1) | VP1 |
| KX420893 | Hu/GII/CA/2016/GII.17/ABEI43 | January, 2016 | GII.17 (cluster 1) | VP1 |
| KX168448 | Hu/GII/HKG/2016/GII.17/CUHK-NS-911 | February, 2016 | GII.17 (cluster 1) | VP1 |
| KX420894 | Hu/GII/CA/2016/GII.17/ABEI68 | February, 2016 | GII.17 (cluster 1) | VP1 |
| LC486758 | Hu/GII/JP/2017/GII.P17-GII.17/FE132 | February, 2017 | GII.17 (cluster 1) | VP1, RdRp |
| LC486760 | Hu/GII/JP/2017/GII.P17-GII.17/FE195 | March, 2017 | GII.17 (cluster 1) | VP1, RdRp |
| LC486759 | Hu/GII/JP/2017/GII.P17-GII.17/FE170 | March, 2017 | GII.17 (cluster 1) | VP1, RdRp |
| LC369217 | Hu/GII/JP/2015/GII.P17-GII.17/GU-57 | February, 2015 | GII.17 (cluster 1) | VP1, RdRp |
| LC369218 | Hu/GII/JP/2015/GII.P17-GII.17/GU-58 | February, 2015 | GII.17 (cluster 1) | VP1, RdRp |
| LC369219 | Hu/GII/JP/2015/GII.P17-GII.17/GU-59 | February, 2015 | GII.17 (cluster 1) | VP1, RdRp |
| LC369220 | Hu/GII/JP/2015/GII.P17-GII.17/GU-60 | February, 2015 | GII.17 (cluster 1) | VP1, RdRp |
| LC369225 | Hu/GII/JP/2015/GII.P17-GII.17/HI-82 | March, 2015 | GII.17 (cluster 1) | VP1, RdRp |
| LC369228 | Hu/GII/JP/2014/GII.P17-GII.17/MI-77 | December, 2014 | GII.17 (cluster 1) | VP1, RdRp |
| LC369229 | Hu/GII/JP/2014/GII.P17-GII.17/MI-79 | December, 2014 | GII.17 (cluster 1) | VP1, RdRp |
| LC369230 | Hu/GII/JP/2014/GII.P17-GII.17/MI-80 | December, 2014 | GII.17 (cluster 1) | VP1, RdRp |
| LC369231 | Hu/GII/JP/2015/GII.P17-GII.17/MI-81 | January, 2015 | GII.17 (cluster 1) | VP1, RdRp |
| LC369232 | Hu/GII/JP/2015/GII.P17-GII.17/MI-83 | January, 2015 | GII.17 (cluster 1) | VP1, RdRp |
| LC369233 | Hu/GII/JP/2015/GII.P17-GII.17/MI-84 | January, 2015 | GII.17 (cluster 1) | VP1, RdRp |
| LC369234 | Hu/GII/JP/2015/GII.P17-GII.17/MI-85 | January, 2015 | GII.17 (cluster 1) | VP1, RdRp |
| LC369235 | Hu/GII/JP/2015/GII.P17-GII.17/MI-86 | January, 2015 | GII.17 (cluster 1) | VP1, RdRp |
| LC369236 | Hu/GII/JP/2015/GII.P17-GII.17/MI-87 | January, 2015 | GII.17 (cluster 1) | VP1, RdRp |
| LC369237 | Hu/GII/JP/2015/GII.P17-GII.17/MI-88 | January, 2015 | GII.17 (cluster 1) | VP1, RdRp |
| LC369238 | Hu/GII/JP/2015/GII.P17-GII.17/MI-89 | January, 2015 | GII.17 (cluster 1) | VP1, RdRp |
| LC369239 | Hu/GII/JP/2015/GII.P17-GII.17/MI-90 | February, 2015 | GII.17 (cluster 1) | VP1, RdRp |
| LC369240 | Hu/GII/JP/2015/GII.P17-GII.17/MI-91 | February, 2015 | GII.17 (cluster 1) | VP1, RdRp |
| LC369243 | Hu/GII/JP/2015/GII.P17-GII.17/MI-94 | February, 2015 | GII.17 (cluster 1) | VP1, RdRp |
| LC369245 | Hu/GII/JP/2015/GII.P17-GII.17/SA-145 | January, 2015 | GII.17 (cluster 1) | VP1, RdRp |
| LC369247 | Hu/GII/JP/2015/GII.P17-GII.17/SA-148 | February, 2015 | GII.17 (cluster 1) | VP1, RdRp |
| LC369255 | Hu/GII/JP/2015/GII.P17-GII.17/TO-146 | February, 2015 | GII.17 (cluster 1) | VP1, RdRp |
| LC369257 | Hu/GII/JP/2015/GII.P17-GII.17/TO-148 | March, 2015 | GII.17 (cluster 1) | VP1, RdRp |
| KX168437 | Hu/GII/HKG/2015/GII.17/CUHK-NS-767 | October, 2015 | GII.17 (cluster 1) | VP1 |
| LC486762 | Hu/GII/JP/2015/GII.P17-GII.17/KS-19-2 | October, 2015 | GII.17 (cluster 1) | VP1, RdRp |
| LC369227 | Hu/GII/JP/2015/GII.P17-GII.17/KA-67 | April, 2015 | GII.17 (cluster 1) | VP1, RdRp |
| LC369252 | Hu/GII/JP/2015/GII.P17-GII.17/SA-165 | October, 2015 | GII.17 (cluster 1) | VP1, RdRp |
| KU557797 | Hu/GII/CN/2014/GII.17/GD-GZ/10-0106 | December, 2014 | GII.17 (cluster 1) | VP1 |

Collected samples in this study are shown by red characters.

| Accession numbers | Strains | Collection dates | Genotypes  (clusters) | Target genes |
| --- | --- | --- | --- | --- |
| KU557859 | Hu/GII/CN/2015/GII.17/GD-DG/34-0576 | March, 2015 | GII.17 (cluster 1) | VP1 |
| KU561230 | Hu/GII/HKG/2015/GII.17/CUHK-NS-504 | January, 2015 | GII.17 (cluster 1) | VP1 |
| KY424349 | Hu/GII/US/2014/GII.P17-GII.17/GaithersburgD7 | December, 2014 | GII.17 (cluster 1) | VP1, RdRp |
| KR083017 | Hu/GII/US/2014/GII.P17-GII.17/Gaithersburg | November, 2014 | GII.17 (cluster 1) | VP1, RdRp |
| KT970376 | Hu/GII/CN/2015/GII.P17-GII.17/Guangzhou/GZ2015-L343 | January, 2015 | GII.17 (cluster 1) | VP1, RdRp |
| KX424647 | Hu/GII/NL/2015/GII.P17-GII.17/Almere278 | May, 2015 | GII.17 (cluster 1) | VP1 |
| KX424649 | Hu/GII/NL/2015/GII.P17-GII.17/Heemskerk337 | November, 2015 | GII.17 (cluster 1) | VP1 |
| KX424650 | Hu/GII/NL/2015/GII.P17-GII.17/Heemskerk336 | November, 2015 | GII.17 (cluster 1) | VP1 |
| KU557886 | Hu/GII/CN/2015/GII.17/GD-ZH/7-0050 | January, 2015 | GII.17 (cluster 1) | VP1 |
| KU557887 | Hu/GII/CN/2015/GII.17/GD-ZH/7-0056 | January, 2015 | GII.17 (cluster 1) | VP1 |
| KU557888 | Hu/GII/CN/2015/GII.17/GD-ZH/7-0057 | January, 2015 | GII.17 (cluster 1) | VP1 |
| KX168444 | Hu/GII/HKG/2016/GII.17/CUHK-NS-896 | February, 2016 | GII.17 (cluster 1) | VP1 |
| KU557881 | Hu/GII/CN/2015/GII.17/GD-ZH/5-0032 | January, 2015 | GII.17 (cluster 1) | VP1 |
| KX134669 | Hu/GII/SI/2015/GII.17/Ljubljana1758 | July, 2015 | GII.17 (cluster 1) | VP1 |
| MF172096 | Hu/GII/US/2016/GII.17/USA-9166 | January, 2016 | GII.17 (cluster 1) | VP1 |
| KY424350 | Hu/GII/US/2014/GII.P17-GII.17/GaithersburgD14 | December, 2014 | GII.17 (cluster 1) | VP1, RdRp |
| KU561239 | Hu/GII/HKG/2015/GII.17/CUHK-NS-583 | February, 2015 | GII.17 (cluster 1) | VP1 |
| KU557807 | Hu/GII/CN/2014/GII.17/GD-QY/15-1649 | December, 2014 | GII.17 (cluster 1) | VP1 |
| KU557808 | Hu/GII/CN/2014/GII.17/GD-QY/15-1650 | December, 2014 | GII.17 (cluster 1) | VP1 |
| KU561235 | Hu/GII/HKG/2015/GII.17/CUHK-NS-546 | January, 2015 | GII.17 (cluster 1) | VP1 |
| KU557869 | Hu/GII/CN/2015/GII.17/GD-HZ/4-0017 | January, 2015 | GII.17 (cluster 1) | VP1 |
| KU557870 | Hu/GII/CN/2015/GII.17/GD-HZ/4-0018 | January, 2015 | GII.17 (cluster 1) | VP1 |
| KU557871 | Hu/GII/CN/2015/GII.17/GD-HZ/4-0019 | January, 2015 | GII.17 (cluster 1) | VP1 |
| LC148854 | Hu/GII/JP/2016/GII.17/Osaka15-428 | January, 2016 | GII.17 (cluster 1) | VP1 |
| KX420895 | Hu/GII/CA/2016/GII.17/ABEI69 | February, 2016 | GII.17 (cluster 1) | VP1 |
| KU561246 | Hu/GII/HKG/2015/GII.17/CUHK-NS-679 | June, 2015 | GII.17 (cluster 1) | VP1 |
| MF172092 | Hu/GII/US/2015/GII.17/USA-7356 | May, 2015 | GII.17 (cluster 1) | VP1 |
| MF172094 | Hu/GII/US/2015/GII.17/USA-7425 | September, 2015 | GII.17 (cluster 1) | VP1 |
| KR052020 | Hu/GII/TW/2015/GII.P17-GII.17/Changhua/15-AH-1 | February, 2015 | GII.17 (cluster 1) | VP1, RdRp |
| MF172095 | Hu/GII/US/2016/GII.17/USA-8986 | January, 2016 | GII.17 (cluster 1) | VP1 |
| KU561227 | Hu/GII/HKG/2014/GII.17/CUHK-NS-484 | December, 2014 | GII.17 (cluster 1) | VP1 |
| LC369246 | Hu/GII/JP/2015/GII.P17-GII.17/SA-146 | January, 2015 | GII.17 (cluster 1) | VP1, RdRp |
| LC486763 | Hu/GII/JP/2015/GII.P17-GII.17/KS-19-3 | November, 2015 | GII.17 (cluster 1) | VP1, RdRp |
| MF172093 | Hu/GII/US/2015/GII.17/USA-7424 | September, 2015 | GII.17 (cluster 1) | VP1 |
| LC369258 | Hu/GII/JP/2015/GII.P17-GII.17/SA-169 | October, 2015 | GII.17 (cluster 1) | VP1, RdRp |
| LC486769 | Hu/GII/JP/2017/GII.P17-GII.17/KS-19-9 | March, 2017 | GII.17 (cluster 1) | VP1, RdRp |

Collected samples in this study are shown by red characters.

| Accession numbers | Strains | Collection dates | Genotypes  (clusters) | Target genes |
| --- | --- | --- | --- | --- |
| LC369256 | Hu/GII/JP/2015/GII.P17-GII.17/TO-147 | March, 2015 | GII.17 (cluster 1) | VP1, RdRp |
| KX168452 | Hu/GII/HKG/2016/GII.17/CUHK-NS-928 | March, 2016 | GII.17 (cluster 1) | VP1 |
| KU557875 | Hu/GII/CN/2015/GII.17/GD-QY/45-1731 | November, 2015 | GII.17 (cluster 1) | VP1 |
| KU557876 | Hu/GII/CN/2015/GII.17/GD-QY/45-1732 | November, 2015 | GII.17 (cluster 1) | VP1 |
| KU557877 | Hu/GII/CN/2015/GII.17/GD-QY/45-1734 | November, 2015 | GII.17 (cluster 1) | VP1 |
| KU557878 | Hu/GII/CN/2015/GII.17/GD-QY/45-1737 | November, 2015 | GII.17 (cluster 1) | VP1 |
| KU557879 | Hu/GII/CN/2015/GII.17/GD-QY/45-1738 | November, 2015 | GII.17 (cluster 1) | VP1 |
| KU557880 | Hu/GII/CN/2015/GII.17/GD-GZ/47-1807 | November, 2015 | GII.17 (cluster 1) | VP1 |
| KU557883 | Hu/GII/CN/2015/GII.17/GD-GZ/51-1818 | November, 2015 | GII.17 (cluster 1) | VP1 |
| KU557884 | Hu/GII/CN/2015/GII.17/GD-GZ/52-1819 | November, 2015 | GII.17 (cluster 1) | VP1 |
| KX168456 | Hu/GII/HKG/2016/GII.17/CUHK-NS-942 | March, 2016 | GII.17 (cluster 1) | VP1 |
| KP902588 | Hu/GII/HKG/2015/GII.17/CUHK-NS-565 | January, 2015 | GII.17 (cluster 1) | VP1 |
| KU561237 | Hu/GII/HKG/2015/GII.17/CUHK-NS-576 | January, 2015 | GII.17 (cluster 1) | VP1 |
| KU557827 | Hu/GII/CN/2014/GII.17/GD-JM/20-1694 | December, 2014 | GII.17 (cluster 1) | VP1 |
| KU557828 | Hu/GII/CN/2014/GII.17/GD-JM/20-1695 | December, 2014 | GII.17 (cluster 1) | VP1 |
| KU557786 | Hu/GII/CN/2014/GII.P17-GII.17/GD-JM/1711 | December, 2014 | GII.17 (cluster 1) | VP1, RdRp |
| KU561242 | Hu/GII/HKG/2015/GII.17/CUHK-NS-610 | February, 2015 | GII.17 (cluster 1) | VP1 |
| KU561238 | Hu/GII/HKG/2015/GII.17/CUHK-NS-580 | February, 2015 | GII.17 (cluster 1) | VP1 |
| KX424648 | Hu/GII/NL/2015/GII.P17-GII.17/Almere279 | May, 2015 | GII.17 (cluster 1) | VP1 |
| KX134670 | Hu/GII/SI/2015/GII.17/Ljubljana1962 | August, 2015 | GII.17 (cluster 1) | VP1 |
| LC486770 | Hu/GII/JP/2017/GII.P17-GII.17/KS-19-10 | October, 2017 | GII.17 (cluster 1) | VP1, RdRp |
| KU555841 | Hu/GII/HKG/2016/GII.17/CUHK-NS-864 | January, 2016 | GII.17 (cluster 1) | VP1 |
| LC318748 | Hu/GII/JP/2016/GII.P17-GII.17/OsakaFB315 | December, 2016 | GII.17 (cluster 1) | VP1, RdRp |
| KX024652 | Hu/GII/HUN/2015/GII.P17-GII.17/HUN5737 | October, 2015 | GII.17 (cluster 1) | VP1 |
| KX346705 | Hu/GII/TH/2015/GII.17/B2194 | September, 2015 | GII.17 (cluster 1) | VP1 |
| KX346700 | Hu/GII/TH/2015/GII.17/B2306 | October, 2015 | GII.17 (cluster 1) | VP1 |
| KX346704 | Hu/GII/TH/2015/GII.17/B2459 | December, 2015 | GII.17 (cluster 1) | VP1 |
| KX346702 | Hu/GII/TH/2015/GII.17/B2387 | November, 2015 | GII.17 (cluster 1) | VP1 |
| KX346703 | Hu/GII/TH/2015/GII.17/B2395 | November, 2015 | GII.17 (cluster 1) | VP1 |
| KX346701 | Hu/GII/TH/2015/GII.17/B2316 | November, 2015 | GII.17 (cluster 1) | VP1 |
| KX346699 | Hu/GII/TH/2014/GII.17/B1995 | October, 2014 | GII.17 (cluster 1) | VP1 |
| KT732276 | Hu/GII/TW/2015/GII.P17-GII.17/15-EN-9 | July, 2015 | GII.17 (cluster 1) | RdRp |
| KT732275 | Hu/GII/TW/2015/GII.P17-GII.17/15-EN-10 | July, 2015 | GII.17 (cluster 1) | RdRp |
| KT906669 | Hu/GII/TW/2014/GII.P17-GII.17/14-BW-3 | December, 2014 | GII.17 (cluster 1) | RdRp |
| KT970371 | Hu/GII/CN/2015/GII.P17-GII.17Guangzhou/GZ2015-L324 | January, 2015 | GII.17 (cluster 1) | RdRp |
| KU557790 | Hu/GII/CN/2014/GII.P17-GII.17/GD-MM/1548 | November, 2014 | GII.17 (cluster 1) | VP1, RdRp |

Collected samples in this study are shown by red characters.

| Accession numbers | Strains | Collection dates | Genotypes  (clusters) | Target genes |
| --- | --- | --- | --- | --- |
| KT906671 | Hu/GII/TW/2014/GII.P17-GII.17/14-BS-1 | December, 2014 | GII.17 (cluster 1) | RdRp |
| KT970375 | Hu/GII/CN/2015/GII.P17-GII.17/Guangzhou/GZ2015-L340 | January, 2015 | GII.17 (cluster 1) | RdRp |
| KT906670 | Hu/GII/TW/2014/GII.P17-GII.17/14-BQ-2 | December, 2014 | GII.17 (cluster 1) | RdRp |
| LC486739 | Hu/GII/JP/2015/GII.P17-GII.17/IB-N14-711 | January, 2015 | GII.17 (cluster 1) | VP1, RdRp |
| LC486740 | Hu/GII/JP/2015/GII.P17-GII.17/IB-N14-792 | February, 2015 | GII.17 (cluster 1) | VP1, RdRp |
| LC486741 | Hu/GII/JP/2015/GII.P17-GII.17/IB-N15-461 | December, 2015 | GII.17 (cluster 1) | VP1, RdRp |
| LC486742 | Hu/GII/JP/2016/GII.P17-GII.17/IB-N16-815 | December, 2016 | GII.17 (cluster 1) | VP1, RdRp |
| LC486743 | Hu/GII/JP/2017/GII.P17-GII.17/IB-N16-981 | March, 2017 | GII.17 (cluster 1) | VP1, RdRp |
| LC486744 | Hu/GII/JP/2017/GII.P17-GII.17/IB-N17-115 | June, 2017 | GII.17 (cluster 1) | VP1, RdRp |
| LC486745 | Hu/GII/JP/2017/GII.P17-GII.17/IB-N17-194 | August, 2017 | GII.17 (cluster 1) | VP1, RdRp |
| LC486753 | Hu/GII/JP/2016/GII.P17-GII.17/YG-4 | March, 2016 | GII.17 (cluster 1) | VP1, RdRp |
| LC486754 | Hu/GII/JP/2016/GII.P17-GII.17/YG-5 | April, 2016 | GII.17 (cluster 1) | VP1, RdRp |
| LC486755 | Hu/GII/JP/2017/GII.P17-GII.17/YG-6 | February, 2017 | GII.17 (cluster 1) | VP1, RdRp |
| LC486756 | Hu/GII/JP/2017/GII.P17-GII.17/HI-S1786 | July, 2017 | GII.17 (cluster 1) | VP1, RdRp |
| LC486757 | Hu/GII/JP/2017/GII.P17-GII.17/HI-S1809 | November, 2017 | GII.17 (cluster 1) | VP1, RdRp |
| LC486750 | Hu/GII/JP/2016/GII.P17-GII.17/AI-28 | April, 2016 | GII.17 (cluster 1) | VP1, RdRp |
| LC486747 | Hu/GII/JP/2015/GII.P17-GII.17/AI-F1 | April, 2015 | GII.17 (cluster 1) | VP1, RdRp |
| LC486746 | Hu/GII/JP/2016/GII.P17-GII.17/AI-F1 | April, 2016 | GII.17 (cluster 1) | VP1, RdRp |
| LC486748 | Hu/GII/JP/2016/GII.P17-GII.17/AI-F4 | April, 2016 | GII.17 (cluster 1) | VP1, RdRp |
| LC486752 | Hu/GII/JP/2016/GII.P17-GII.17/AI-F289 | March, 2016 | GII.17 (cluster 1) | VP1, RdRp |
| LC486749 | Hu/GII/JP/2016/GII.P17-GII.17/AI-F15 | April, 2016 | GII.17 (cluster 1) | VP1, RdRp |
| LC486751 | Hu/GII/JP/2015/GII.P17-GII.17/AI-F101 | December, 2015 | GII.17 (cluster 1) | VP1, RdRp |
| AB684681 | Hu/GII/JP/1976/GII.P3-GII.17/Tokyo/27-3 | March, 1976 | GII.17  (ancestral strain of clusters 1 and 2) (cluster 3) | VP1 |
| AY502009 | Hu/GII/US/2002/GII.P16-GII.17/CS-E1 | 2002 | GII.17 (cluster 4) | VP1 |
| KJ196286 | Hu/GII/JP/2002/GII.P16-GII.17/Saitama/T87 | 2002 | GII.17 (cluster 4) | VP1 |
| KC597139 | Hu/GII/GUF/1978/GII.P4-GII.17/C142 | July, 1978 | GII.17 (cluster 5) | VP1 |
| KT589391 | Hu/GII/HKG/2015/GII.Pe-GII.17/CUHK-NS-682 | June, 2015 | GII.17 (cluster 6) | VP1 |
| DQ438972 | Hu/GII/US/2005/GII.17/Katrina-17 | 2005 | GII.17 (cluster 7) | VP1 |

Collected samples in this study are shown by red characters.

Table S2. PCR and sequencing primers for full-lengths of *VP1* and *RdRp* regions in the GII.P17-GII.17 strains

| Primers | Polarity | Sequence (5' to 3') | Target genes | Methods | PCR conditions | References |
| --- | --- | --- | --- | --- | --- | --- |
| GII.P17-PCR-1F | + | GGACAACAGGGGAGCTGAT | RdRp | PCR | 45°C 30 min  ↓  94°C 2 min  ↓  98°C 30 sec  ↓  55°C 30 sec  ↓  68°C 90 sec  ↓  68°C 7 min  45 cycles | This study |
| G2SKR | - | CCRCCNGCATRHCCRTTRTACAT | RdRp | PCR |  | Kojima et al., 2002 |
| GII.P17-Seq-1F | + | TTCTCACTGGCTCAAATGCTAA | RdRp | Sequencing |  | This study |
| GII.P17-Seq-2F | + | CCCCCTCAAAAGTGGTCATAT | RdRp | Sequencing |  | This study |
| GII.P17-Seq-3F | + | CAGGTACAGGTACCACTATGATGC | RdRp | Sequencing |  | This study |
| GII.P17-Seq-4F | + | ATGGGTTGACTTTCCTCCG | RdRp | Sequencing |  | This study |
| COG2F | + | CARGARBCNATGTTYAGRTGGATGAG | RdRp | Sequencing |  | Motomura et al., 2008 |
| COG2F | + | CARGARBCNATGTTYAGRTGGATGAG | VP1 | PCR | 45°C 30 min  ↓  94°C 2 min  ↓  98°C 30 sec  ↓  55°C 30 sec  ↓  68°C 90 sec  ↓  68°C 7 min  45 cycles | Motomura et al., 2008 |
| GII.17-PCR-5R | - | CTGTTTTAGGGCTATCATTTCAGATT | VP1 | PCR |  | This study |
| G2SKR | - | CCRCCNGCATRHCCRTTRTACAT | VP1 | Sequencing |  | Kojima et al., 2002 |
| G2SKF | + | CNTGGGAGGGCGATCGCAA | VP1 | Sequencing |  | Motomura et al., 2008 |
| GII.17-Seq-1F | + | TCTTGAACCAATTATGATCCCAC | VP1 | Sequencing |  | This study |
| GII.17-Seq-2F | + | GTGCATTCAGAGGACGGG | VP1 | Sequencing |  | This study |
| GII.17-Seq-3F | + | GCTCCAAATTTYCCTGGTGA | VP1 | Sequencing |  | This study |
| GII.17-Seq-4F | + | ATCCTCTTGTTGTTCCGGC | VP1 | Sequencing |  | This study |

**References**

Kojima, S., Kageyama, T., Fukushi, S., Hoshino, F.B., Shinohara, M., Uchida, K., et al. (2002). Genogroup-specific PCR primers for detection of Norwalk-like viruses. J. Virol. Methods 100, 107-114.

Motomura, K., Oka, T., Yokoyama, M., Nakamura, H., Mori, H., Ode, H., et al. (2008). Identification of monomorphic and divergent haplotypes in the 2006-2007 norovirus GII/4 epidemic population by genomewide tracing of evolutionary history. J. Virol. 82, 11247-11262.
